# Chloro­cobalt complexes with pyridyl­ethyl-derived di­aza­cyclo­alkanes

**DOI:** 10.1107/S2056989022001220

**Published:** 2022-02-15

**Authors:** Anthony W. Addison, Stephen J. Jaworski, Jerry P. Jasinski, Mark M. Turnbull, Fan Xiao, Matthias Zeller, Molly A. O’Connor, Elizabeth A. Brayman

**Affiliations:** a Department of Chemistry, Drexel University, Philadelphia, PA 19104, USA; b Department of Chemistry, Keene State College, Keene, NH 03435, USA; c Carlson School of Chemistry and Biochemistry, Clark University, 950 Main St., Worcester, MA 01610, USA; d Department of Chemistry, Purdue University, 560 Oval Drive, West Lafayette, IN, 47907-2084, USA

**Keywords:** crystal structure, cobalt, magnetism, ZFS, piperazines, DFT, NIR spectra, electronic spectra

## Abstract

Syntheses are described for the blue/purple complexes of cobalt(II) chloride with the tetra­dentate ligands 1,4-bis­[2-(pyridin-2-yl)eth­yl]piperazine (Ppz), 1,4-bis­[2-(pyridin-2-yl)eth­yl]homopiperazine (Phpz), *trans*-2,5-dimethyl-1,4-bis­[2-(pyridin-2-yl)eth­yl]piperazine (Pdmpz) and tridentate 4-methyl-1-[2-(pyridin-2-yl)eth­yl]homopiperazine (Pmhpz). The CoCl_2_ complexes with Ppz, namely, {μ-1,4-bis­[2-(pyridin-2-yl)eth­yl]piperazine}bis­[di­chlorido­cobalt(II)], [Co_2_Cl_4_(C_18_H_24_N_4_)] or Co_2_(Ppz)Cl_4_, and Pdmpz (structure not reported as X-ray quality crystals were not obtained), are shown to be dinuclear, with the ligands bridging the two tetra­hedrally coordinated CoCl_2_ units. Co_2_(Ppz)Cl_4_ and {di­chlorido­{4-methyl-1-[2-(pyridin-2-yl)eth­yl]-1,4-di­aza­cyclo­hepta­ne}cobalt(II) [CoCl_2_(C_13_H_21_N_3_)] or Co(Pmhpz)Cl_2_, crystallize in the monoclinic space group *P*2_1_/*n*, while crystals of the penta­coordinate mono­chloro chelate 1,4-bis­[2-(pyr­id­in-2-yl)eth­yl]piperazine}chlorido­cobalt(II) perchlorate, [CoCl(C_18_H_24_N_4_)]ClO_4_ or [Co(Ppz)Cl]ClO_4_, are also monoclinic (*P*2_1_). The complex {1,4-bis­[2-(pyridin-2-yl)eth­yl]-1,4-di­aza­cyclo­hepta­ne}di­chlorido­cobalt(II) [CoCl_2_(C_19_H_26_N_4_)] or Co(Phpz)Cl_2_ (*P*  ) is mononuclear, with a penta­coordinated Co^II^ ion, and entails a Phpz ligand acting in a tridentate fashion, with one of the pyridyl moieties dangling and non-coordinated; its displacement by Cl^−^ is attributed to the solvophobicity of Cl^−^ toward MeOH. The penta­coordinate Co atoms in Co(Phpz)Cl_2_, [Co(Ppz)Cl]^+^ and Co(Pmhpz)Cl_2_ have substantial trigonal–bipyramidal character in their stereochemistry. Visible- and near-infrared-region electronic spectra are used to differentiate the two types of coordination spheres. TDDFT calculations suggest that the visible/NIR region transitions contain contributions from MLCT and LMCT character, as well as their expected *d–d* nature. For Co(Pmhpz)Cl_2_ and Co(Phpz)Cl_2_, variable-temperature magnetic susceptibility data were obtained, and the observed decreases in moment with decreasing temperature were modelled with a zero-field-splitting approach, the *D* values being +28 and +39 cm^−1^, respectively, with the *S* = 1/2 state at lower energy.

## Abstract 2

With cobalt(II) chloride, some piperazine- and homo*-*piperazine-derived ligands yield tetra- or penta­coordinate complexes. Observed variations in coordination number are ascribed as being related to chloride solvophobicity. Optical spectra are presented, while magnetism measurements indicate governance of the magnetism by zero-field splitting of the cobalt ion.

## Chemical context

**Figure 1:**
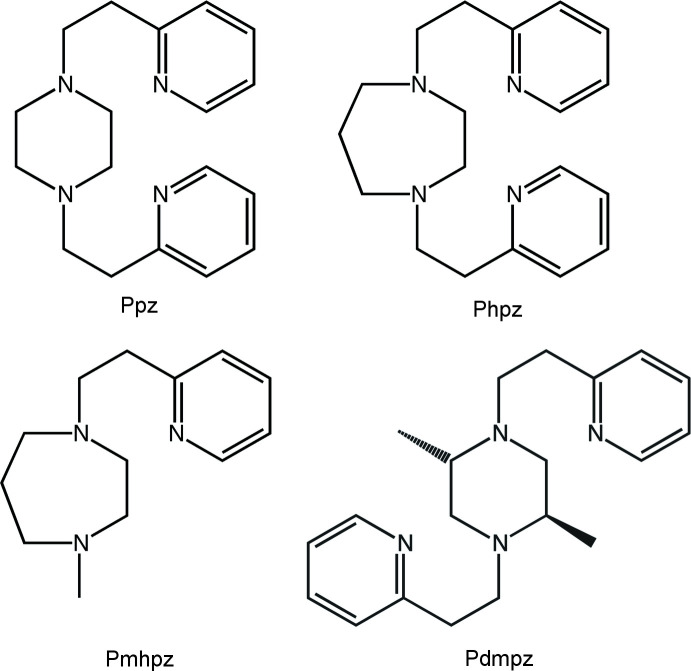
Ligands employed in this work.

Pyridyl­ethyl­ation of amines has previously been used to prepare a variety of chelating agents (Phillip *et al.*, 1970 ▸; Profft & Georgi, 1961 ▸; Profft & Lojack 1962 ▸; Gray *et al.*, 1960 ▸; Kryatov *et al.*, 2002 ▸; Kryatova *et al.*, 2012 ▸; Marsich *et al.*, 1998 ▸; Karlin *et al.*, 1984 ▸; Anandababu *et al.*, 2020 ▸; Muthuramalingam *et al.*, 2019*a* ▸,*b* ▸), with an original driver being the generation of biomimetic mol­ecules (Karlin *et al.*, 1984 ▸). Examples immediately relevant to the present work (Fig. 1 ▸) include 1,4-bis­[2′-(2′′-pyridyl­eth­yl)]piperazine (Ppz) and 1,4-bis­[2′-(2′′-pyridyl­eth­yl)]homo-piperazine, Phpz. Phpz was first prepared by Schmidt *et al.* (2013 ▸), while Jain and coworkers reported Ppz in 1967 (Jain *et al.*, 1967 ▸). For Ppz, both copper(II) (Mautner *et al.*, 2008 ▸, 2009 ▸; O’Connor *et al.*, 2012 ▸) and nickel(II) (O’Connor *et al.*, 2012 ▸) complexes have been described. In the case of Phpz, there are reports of copper(II) complexes (O’Connor *et al.*, 2012 ▸), including their application as oxidation catalysts (Muthuramalingam *et al.*, 2017 ▸, 2020 ▸). In addition, nickel(II) complexes of Phpz have been studied as catalysts (Muthuramalingam *et al.*, 2019*a* ▸,*b* ▸) as has a recent cobalt(II) complex (Anandababu *et al.*, 2020 ▸). For Pmhpz, copper and nickel complexes have been characterized (O’Connor *et al.*, 2012 ▸), and Muthuramalingam and co-workers have recently examined oxidative catalysis by copper complexes including that of Pmhpz (Muthuramalingam *et al.*, 2021 ▸), but there appears to be only the single prior report of Pdmpz (O’Connor *et al.*, 2012 ▸). Four structures are described here. X-ray quality crystals of the Pdmzp complex were not obtained. 
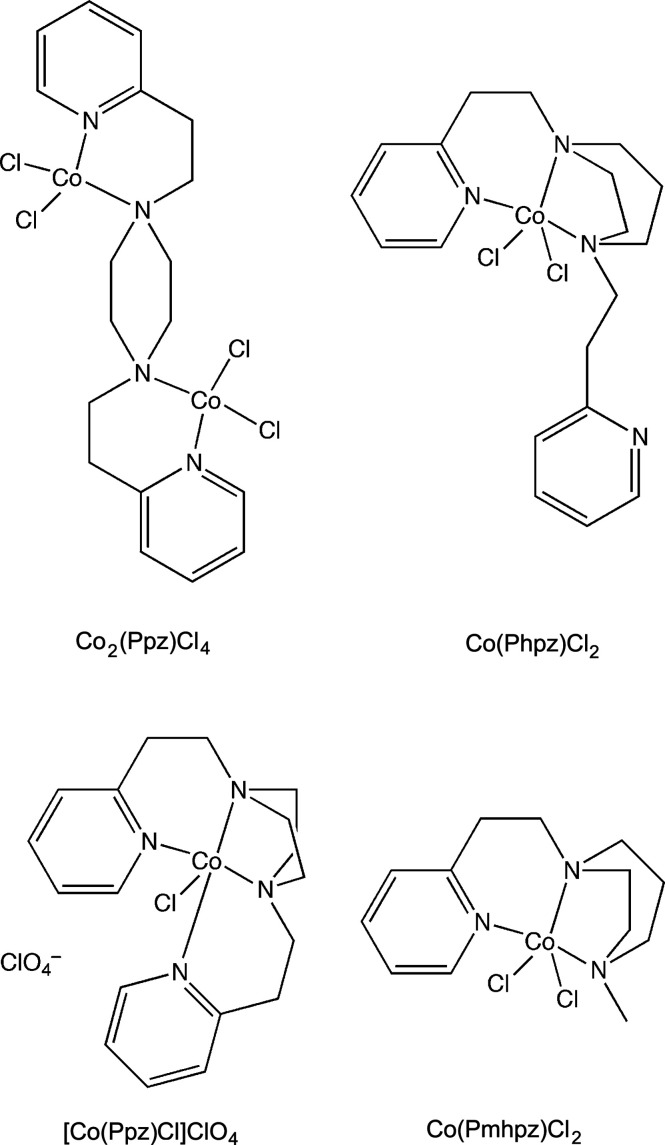


## Structural commentary

The structures are not all entirely what was originally expected, based on previous work with these types of ligands. The Co–N(Pyr) bond lengths (Tables 1 ▸–4 ▸ ▸ ▸) range from 2.03 to 2.16 Å, which is within the usual span (Orpen *et al.*, 1989 ▸), while the Co—Cl distances average 2.28 ± 0.03 Å, which is again common for cobalt(II) (Orpen *et al.*, 1989 ▸). The Co—N_amine_ bond lengths are generally longer than the Co—N_pyridine_ ones, and quite variable (*vide infra*), with an average of 2.154 Å and covering a 0.153 Å range. The distances are unexceptional for Co^II^ to tertiary amine linkages (Orpen *et al.*, 1989 ▸), and indeed tertiary amine nitro­gen atoms in tripodal ligands are often notably more distant from the Co^II^ ion (2.44–3.27 Å; Brewer, 2020 ▸).

**Figure 2:**
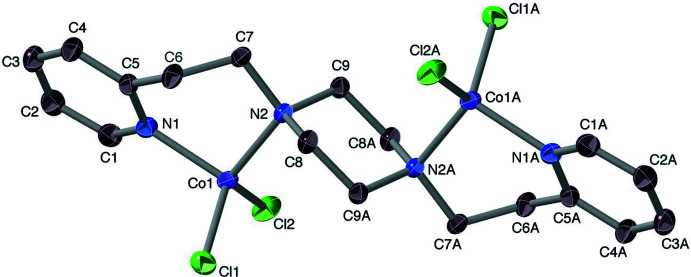
Mol­ecular structure of Co_2_(Ppz)Cl_4_. Ellipsoids are drawn at the 50% level, and for clarity of presentation, H atoms are omitted. The two half-mol­ecules in the structure are symmetry equivalent and are related to the other halves *via* the symmetry operation (1 − *x*, 1 − *y*, 2 − *z*).

For the CoCl_2_-Ppz combination, the dinuclear compound Co_2_(Ppz)Cl_4_ was obtained (Fig. 2 ▸), rather than the mononuclear Co(Ppz)Cl_2_. The asymmetric unit in this *P*2_1_/*n* structure is the half-mol­ecule, related to the mol­ecule’s other corresponding half by an inversion centre.

The piperazine moiety in Co(Ppz)Cl_2_ does not chelate a cobalt ion, but instead bridges between two, so that each tetra­coordinate Co is bound by a piperazine-N atom, a pyridyl-N atom and two chloride ions. The two identical coordination cores have *ω* = 86° (Sakaguchi & Addison, 1979 ▸) and *φ_t_* = 0.07 (Addison *et al.*, 2004 ▸; Yang *et al.*, 2007 ▸), so are fairly close to exactly tetra­hedral in geometry.

**Figure 3:**
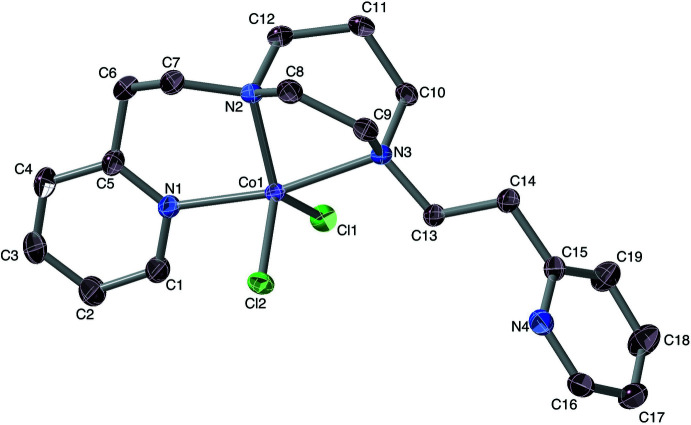
Structure of Co(Phpz)Cl_2_, with its dangling pyridine moiety. The dominant conformer is shown. Ellipsoids are drawn at the 50% level, and for clarity of presentation, H atoms are omitted.

As the same ligand behaves as a straightforward mononucleating quadridentate in the copper and nickel complexes (O’Connor *et al.*, 2012 ▸; Muthuramalingam *et al.*, 2017 ▸, 2019*a* ▸,*b* ▸), this led to the question as to whether the coordination is governed by the ligand bite *vs* the larger ionic radius of Co^2+^ *vs* Cu^2+^/Ni^2+^. This proposal was approached by synthesising the homopiperazine analogue, Phpz, whose ligand has a larger (N2—N2*A*) bite. The compound Co(Phpz)Cl_2_ was indeed obtained as a mononuclear product (Fig. 3 ▸), crystallizing into a *P* 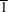 lattice. The structure suffers some disorder, but one conformation is dominant, at 91% (the discussion below refers to that major component of the Co(Phpz)Cl_2_ crystals). However, anti­cipatedly quadridentate Phpz is now seen to act as a tridentate ligand, with the cobalt(II) ion being penta­coordinate.

**Figure 4:**
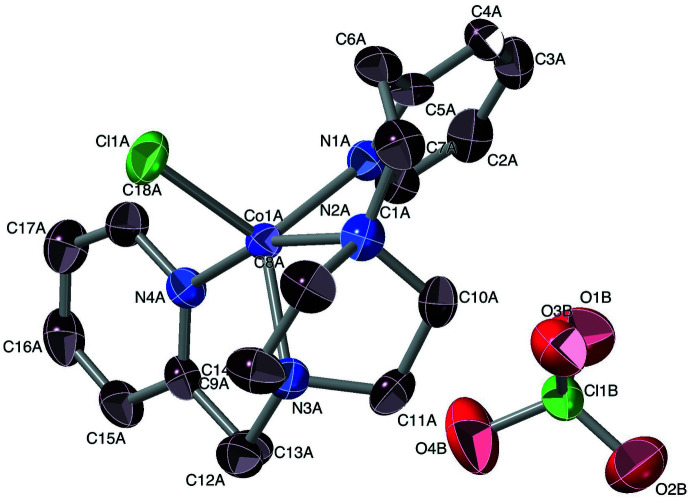
Structural representation of [Co(Ppz)Cl]ClO_4_ (major component). The perchlorate is disordered by a rocking motion along the O2*B*–Cl1*B*–O4*B* direction, which may be related to weak C—H⋯O hydrogen-bonding inter­actions. Ellipsoids are drawn at the 50% level, and for clarity of presentation, H atoms are omitted.

One of the pyridyl­ethyl arms is now in the less-commonly observed dangling mode, pyridine being a consistent protagonist of this phenomenon (Reeves *et al.*, 2014 ▸; Ball *et al.*, 1981 ▸; Rajendiran *et al.*, 2008 ▸; Camerano *et al.*, 2011 ▸; Lonnon *et al.*, 2006 ▸; Palaniandavar *et al.*, 1996 ▸). The core geometry is markedly toward the trigonal–bipyramidal (*τ* = 0.62) (Addison *et al.*, 1984 ▸) with Cl2 acting as the erstwhile reference tetra­gonal axial ligand. The bond from the cobalt ion to the piperazine nitro­gen atom (N3) holding the dangling arm is 0.08 (3) Å longer than the one associated with the coordinated pyridine arm. Inasmuch as the ability of Phpz to act as a tetra­dentate toward Co^II^ has recently been demonstrated in [Co(Phpz)Cl](BPh_4_) (Anandababu *et al.*, 2020 ▸), it is clear that ligand bite is not the sole factor governing the structural outcome in Co(Phpz)Cl_2_. However, all the complexes herein were prepared in non-aqueous solvents – methanol or THF – and we propose that the chloride ion, with its substantial hydration energy, is solvofugic enough to displace a terminal pyridine in a complex involving cobalt(II). We hence prepared the compound of composition [Co(Ppz)Cl]ClO_4_, thus removing a chloride from the binding competition. The resulting structure bears out this hypothesis (Fig. 4 ▸).

[Co(Ppz)Cl)]ClO_4_ crystallizes in the space group *P*2_1_, and entails the [Co(Ppz)Cl)]^+^ cation. This structure has *τ* = 0.65, so is substanti­ally trigonal–bipyramidal in its coordination geometry; the reference axis is Co1*A*–Cl1*A*, and the (pseudo)trigonal axis is N2*A*–Co1*A*–N4*A*. The cation is asymmetric, with non-matching Co—N_pyridine_ bonds of 2.057 (5) and 2.109 (5) Å, while the Co—N_amine_ distances are notably inequivalent, at 2.098 (5) for Co1*A*—N3*A*, but 2.238 (5) Å for Co1*A*—N2*A* – the longest Co—N bond in this set of four compounds. One might note that N3*A* is ‘trigonal–equatorial’, *vs* N2*A* being ‘trigonal–axial’, and suspect that this longer bond betokens an instability that leads to Co_2_(Ppz)Cl_4_. The perchlorate may be involved with quite weak C—H⋯O hydrogen-bonding inter­actions: *e.g.*, C11*A⋯*O3*B*, C13*A*⋯O4*B*, and C11*A*⋯O4*C* are 3.28, 3.46 and 3.60 Å, respectively.

**Figure 5:**
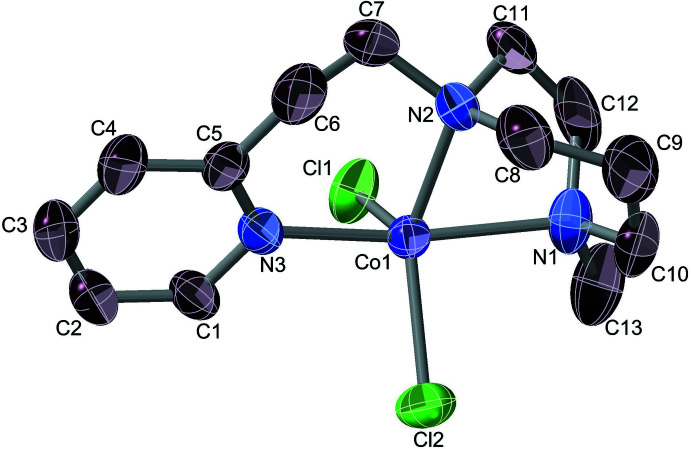
Mol­ecular structure of the complex Co(Pmhpz)Cl­_2_, with the ligand in which a pyridyl arm is replaced by a methyl group. Ellipsoids are drawn at the 50% level, and for clarity of presentation, H atoms are omitted.

In a further experimental essay, we eliminated an otherwise dangling pyridyl arm by replacing it with a methyl group, as in the simpler tridentate ligand Pmhpz. The resulting mol­ecule, Co(Pmhpz)Cl_2_ (Fig. 5 ▸) crystallizes in the *P*2_1_/*n* space group.

The coordination core is somewhat trigonal–bipyramidal, with *τ* = 0.57 and the reference axis being Co1–Cl1. The sole pyridine nitro­gen N3 and the methyl­ated piperazine nitro­gen N1 form the pseudo-trigonal axis. Analogously to the [Co(Ppz)Cl)]^+^ situation, the pseudo-equatorial Co—N2_amine_ bond, at 2.097 (4) Å, is shorter that the Co—N1_amine_ [2.232 (5) Å] and Co—N3_pyridine_ [2.146 (4) Å] bonds in the trigonal directions. One may note that the same axial *vs* equatorial Co–N bond-length relationship also holds for Co(Phpz)Cl_2_, above.

**Figure 6:**
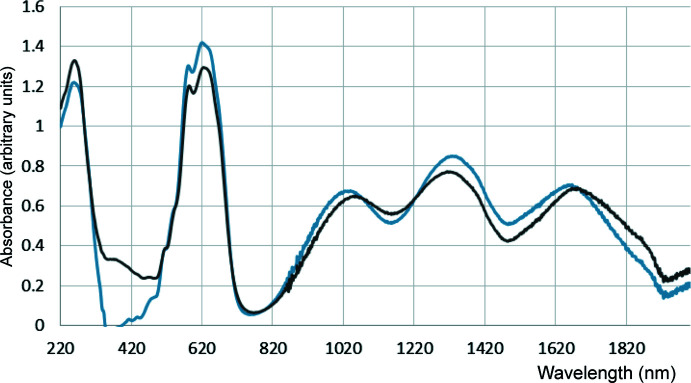
Solid-state diffuse reflectance spectra of [Co_2_(Ppz)Cl_4_] (blue trace) and Co(Pmhpz)Cl_2_ (black trace).

**Electronic spectra: Pseudo­tetra­hedral species:** The essentially identical UV–Vis–NIR spectra for [Co_2_(Ppz)Cl_4_] and Co(Pdmpz)Cl_2_ (Fig. 6 ▸, Table 5 ▸) strongly implicate a tetra­hedral CoN_2_Cl_2_ coordination geometry for the latter, and its constitution as [Co_2_(Pdmpz)Cl_4_] is ultimately confirmed by the elemental analyses (*vide infra*).

**Figure 7:**
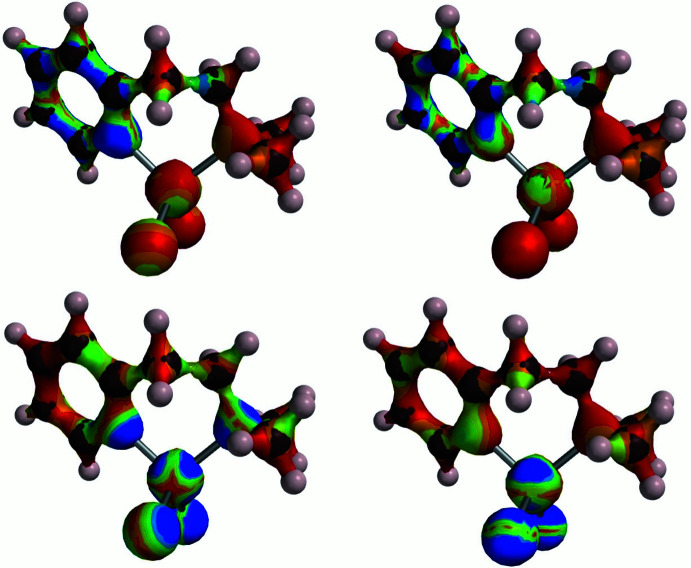
Wavefunction density surface maps of MOs involved in several of the visible-NIR transitions in a CoN_2_Cl_2_ moiety of Co_2_(Ppz)Cl_4_, modelled with a 2-(di­methyl­amino­eth­yl)pyridine ligand. Lower left and right: originating HOMO(−3), HOMO(−4), respectively; upper left and right, the receiving LUMO and LUMO(+1), respectively. Blue indicates highest density. Note the translation of wavefunction density from the CoCl_2_ or CoN_2_Cl_2_ unit to the pyridine ring in the excitations.

Both might also be compared to [Co(Me_4_en)]Cl_2_, which has maxima at *ca* 1670, 1380, 1000, 650 and 580 nm, attributed in a crystal-field model to ^4^ *A*_2_ → ^4^ *T*_1_ (*F*) transitions (the first three) (Lever, 1984 ▸), and the latter two to ^4^ *A*_2_ → ^4^ *T*_1_ (*P*). Though shifted slightly, these maxima are quite similar to the bands for [Co_2_(Ppz)Cl_4_] and [Co_2_(Pdmpz)Cl_4_]. The DFT results for a CoN_2_Cl_2_ chromophore of Co_2_(Ppz)Cl_4_ suggest that even the low-energy transitions involve CT contributions from the CoCl_2_ moiety to the pyridine ring (Fig. 7 ▸).

**Penta­coordinate Systems:** Like [Co_2_(ppz)Cl_4_] and other CoN_2_Cl_2_ chromophores, the roughly trigonal–bipyramidal archetypal CoN_3_Cl_2_ systems Co(Me_5_dien)Cl_2_ and [Co(Et_4_dien)Cl_2_] also have strong ligand-field absorptions in the visible region near 500 and 650 nm, as well as NIR bands at *ca* 2500, 1140, and 950 nm (Ciampolini & Speroni, 1966 ▸; Lever, 1984 ▸). These transitions have been assigned as from ^4^ *A*_2_′ to ^4^ *E*, ^4^ *A*_2_(*P*) and ^4^ *E*(*P*) (Lever, 1984 ▸). More recent examples of CoN_3_Cl_2_ centres (Xiao *et al.*, 2018 ▸) display similarly structured bands with maxima around 650–700 nm. The absorption bands for [Co(Phpz)Cl_2_] resemble those of the above examples to various extents.

**Figure 8:**
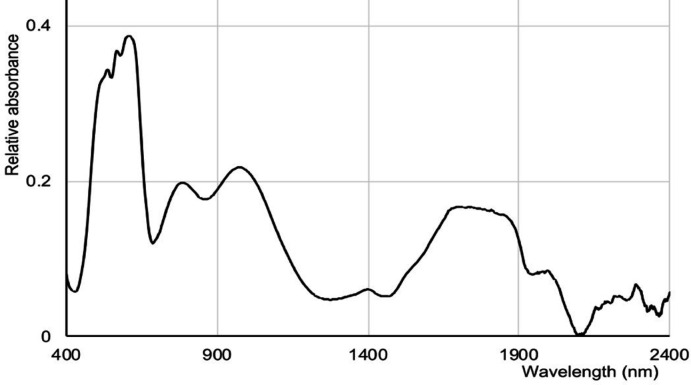
Solid-state Vis–NIR spectrum of [Co(Phpz)Cl_2_].

Figs. 8 ▸ and 9 ▸ show the solid-state spectra of CoPhpzCl_2_ and [Co(Ppz)Cl]ClO_4_, respectively. In comparison with the CoN_2_Cl_2_ cores, one should note the rather different pattern of absorption bands in the NIR. Firstly, the band near 1000 nm appears to be supplanted by two bands, one being near 750 nm, the other around 950 nm. More tellingly, the 1100–1500 nm region, which has clear CoN_2_Cl_2_ maxima near 1300 and 1700 nm, becomes hollowed out, and broader features appear at 1600–1900 nm. The Vis–NIR spectrum (Fig. S9 in the supporting information) of Co(Pmhpz)Cl_2_ is, as expected, similar to that of Co(Phpz)Cl_2_. We do note that the utility of NIR spectroscopy for tetra- and penta­coordinate cobalt(II) complexes, pioneered by Goodgame & Goodgame (1965 ▸) has hardly been widely adopted (Table S1).

**Magnetism analysis**

**Figure 10:**
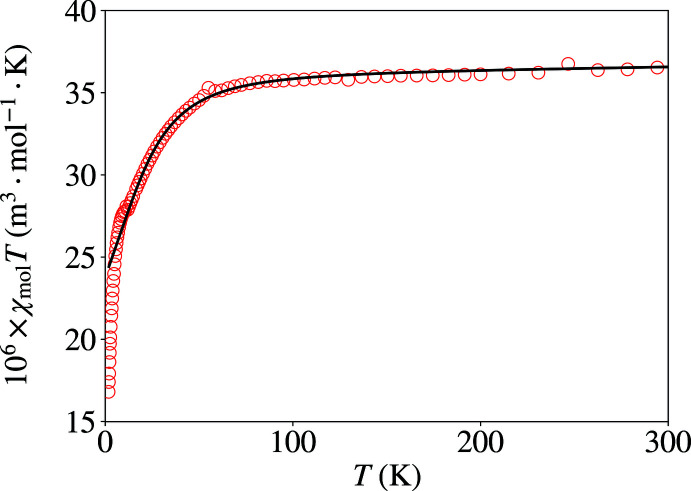
Temperature dependence of χ*T* for Co(Pmhpz)Cl_2_. The solid line is the fit using an exact diagonalization method, between 12.5 and 310 K. (Note that the usual units for molar susceptibility χ have been replaced here by SI units: 1 cm^3^ mol^−1^ = 4π ×10 ^−6^ m^3^ mol^−1^.)

Preliminary data indicated apparently reduced magnetic moments for some samples. However, the structures do not suggest the possibility of any pathway for significant superexchange coupling. Inasmuch as there are penta­coordinate cobalt(II) complexes that have recently been discovered to act as single-ion/single-mol­ecule magnets (SIM/SMM) at reduced temperature (Rechkemmer *et al.*, 2016 ▸; Świtlicka *et al.*, 2018 ▸), we studied the temperature dependence of the magnetic behaviour of powdered samples of Co(Pmhpz)Cl_2_ and Co(Phpz)Cl_2_ (Figs. 10 ▸ and 11 ▸).

The magnetism as a function of temperature and applied field showed no evidence for SMM behaviour. In situations like this, the temperature dependence of the moments has been recognized as being due to zero-field splitting (Nemec *et al.*, 2016 ▸; Cruz *et al.*, 2018 ▸; Boča *et al.*, 1999 ▸; Papánková *et al.*, 2010 ▸; Rajnák *et al.*, 2013 ▸; Żurowska *et al.*, 2008 ▸) (see the supporting information for further discussion). We were able to fit the data through most of the temperature regime and the extracted *D*, *g*_ave_, Δ, *a* and *b* which are listed in Table 6 ▸, *via*:

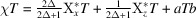

where χ_*x*_ and χ_*z*_ are the longitudinal and transverse modes of the anisotropic responses (Δ = *S_x_*/*S_z_*), *a* is the TIP and *b* the total diamagnetic correction.

Both compounds have a positive axial single-ion anisotropy (SIA) term, and the anisotropy values also confirm the findings as self-consistent (*e.g.* Δ > 1 for positive *D* and Δ < 1 for negative *D*, and larger *D* leads to larger Δ). The *D* and *g*_ave_ values appear to be in the normal ranges; *D* values for Co^II^ do cover a wide range, from *ca* −50 to +100 cm^−1^ (Cruz *et al.*, 2018 ▸; Nemec *et al.*, 2016 ▸). While Co^II^ *g* values intrinsically also cover a wide range, applicable values for fitting ZFS data have been observed to be about 2.0–2.4 (Voronkova *et al.*, 1974 ▸; Baum *et al.*, 2016 ▸; Banci *et al.*, 1980 ▸; Martinelli *et al.*, 1989 ▸). Both compounds here show a faster drop in χ*T* and a distinct kink at temperatures below *ca* 15 K. These features have been seen in several other Co^II^ systems (Żurowska *et al.*, 2008 ▸; Papánková *et al.*, 2010 ▸; Boča *et al.*, 1999 ▸; Rajnák *et al.*, 2013 ▸); however, no definitive accounting for this has been advanced as yet, apart from the not infrequently employed addition of a weak anti­ferromagnetism mean field term.

## Supra­molecular features

There are no true supra­molecular structures formed by the compounds, whose crystal lattices containing individual mol­ecules are defined mainly by weak, non-bonding inter­actions. Along with the absence of any solvation of these crystals, the only hydrogen-bonding inter­actions observed are in [Co(Ppz)Cl]ClO_4_, which has weak C—H⋯O hydrogen-bonds (numerical values are given in the CIF), likely of little energetic consequence.

Some lattice views of the compounds are displayed in the supporting information (Figs. S1–S8).

## Database survey

Closely related compounds with similar *M*(pyridyl­ethyl­piperazine)*X*_2_, *M*(pyridyl­ethyl­piperazine)*X* ^+^, *M*(pyridyl­ethyl­homopiperazine)*X*_2_ or *M*(pyridyl­ethyl­homo­piperazine)*X* ^+^ structures include [Co(Phzp)Cl]BPh_4_ (Anandababu *et al.*, 2020 ▸) and Cu(Dpzp)(NC·N·CN)ClO_4_ (Mautner *et al.*, 2008 ▸).

## Synthesis and crystallization

**Methods**

Chemical ionization mass spectra were obtained on a Thermo-Electron LTQ–FT 7T FT–ICR instrument. UV–visible–near infrared spectra were obtained using PerkinElmer Lambda-35 or Shimadzu UV3600Plus spectrophotometers equipped with integrating spheres for solid-state spectroscopy. Magnetic susceptibility data between 1.8 and 310 K in an applied field of 1 kOe were collected using a Quantum Design MPMS-XL SQUID magnetometer. Crystals were powdered and packed into #3 gel capsules that were placed inside drinking straws attached to the sample rod. The magnetization was measured at 1.8 K as a function of increasing field from zero to five tesla and at selected fields returning to zero. The data were corrected for the contributions from the sample holders (measured independently) and the diamagnetism of the constituent atoms, as estimated using Pascal’s constants (Carlin, 1986 ▸). DFT calculations were performed using the ωB97X-D/6-31G* method on an iMac16,2 with *Spartan-18* software (Wavefunction Inc., Irvine CA, version 1.4.4), while structural diagrams were generated using the *CrystalMaker-10* software and *Preview-10*. Reagents were used as received from TCI America, Sigma–Aldrich, MCB and Fisher Scientific. Elemental microanalyses were by Robertson Microlit Laboratories (Ledgewood, NJ).

Ligands were prepared by adaptions of the solventless method (Addison & Burke, 1981 ▸), typically using a 5–50% excess of 2-vinyl­pyridine plus a catalytic amount of acetic acid, and were then, in effect, purified as the metal complexes (Phillip *et al.*, 1970 ▸); these ligand synthesis reactions are not necessarily stoichiometric or irreversible (Profft & Lojack, 1962 ▸). The procedure is exemplified by:

**1,4-Bis[2-(pyrid-2-yl)eth­yl]piperazine (Ppz):** A mixture of piperazine (0.86 g, 10 mmol), 2-vinyl­pyridine (3.15 g, 30 mmol), and 2 drops of glacial acetic acid was set to react at *ca* 368 K for 14 to 50 h in a capped tube. The reaction mixture was allowed to cool to room temperature, resulting in the formation of a brown solid mass. The mass spectrum indicated Ppz as the dominant component of the solid: *m*/*z* = 297.207, calculated for (C_18_H_24_N_4_+H)^+^, 297.208. The crude ligand was used without purification in the synthesis of the cobalt complexes.

**1,4-Bis[2-(pyridin-2-yl)eth­yl]homopiperazine (Phpz):** From 2-vinyl­pyridine (6.32 g, 60 mmol) and homopiperazine (2.01 g, 20 mmol); crude ligand as a brown mass; *m*/*z* = 311.223, calculated for (C_19_H_26_N_4_+H)^+^, 311.224.

***trans*** **-2,5-Dimethyl-1,4-bis­[2-(pyridin-2-yl)eth­yl]piperazine (Pdmpz):** From *trans*-2,5-di­methyl­piperazine (2.28 g, 20 mmol) and 2-vinyl­pyridine (6.32 g, 60 mmol) as a brown solid mass mingled with white crystals. *m*/*z* = 325.239, calculated for (C_20_H_28_N_4_+H)^+^, 325.239.

**4-Methyl-1-[2-(pyridin-2-yl)eth­yl]homopiperazine (Pmhpz):** *N*-methyl­homopiperazine (1.14 g, 10 mmol) and 2-vinyl­pyridine (1.10 g, 10.5 mmol): heated at the boiling point (*ca* 433 K) for 3 min.; as a viscous brown oil; *m*/*z* = 220.181, calculated for (C_13_H_21_N_3_+H)^+^, 220.181

**Synthesis of cobalt complexes:** The cobalt(II) compounds were mainly prepared by the general method exemplified for [Co_2_(Ppz)Cl­_4_] below, using amounts of crude ligands equivalent to the mol­ecular content of the di­aza­cyclo­alkane used for the ligand synthesis.

**[Co_2_(Ppz)Cl_4_]:** Crude ligand equivalent to 12.0 mmol Ppz, in methanol (30 mL), was combined with 10.0 mmol (6.5 mL of 1.54 *M*) methano­lic cobalt(II) chloride hydrate solution. Deep-blue crystals deposited, which were filtered off and recrystallized from nitro­methane. The mass spectrum showed several elucidatory peaks, including *m*/*z* = 518.975 for (*M* − Cl)^+^ = Co_2_PpzCl_3_^+^ (calculated 518.973) as well as *m*/*z* = 426.079 (CoPpzCl_2_H^+^, calculated 426.078) and *m*/*z* = 390.102 (CoPpzCl^+^, calculated 390.102). Analysis C,H,N: found %, C 39.08, H 4.10, N 9.70; calculated for C_18_H_24_Cl_4_Co_2_N_4_: C 38.88, H 4.35, N 10.08.

**[Co(Phpz)Cl­_2_]:** In this case, the CoCl_2_ solution was added to the ligand in tetra­hydro­furan. When the solution was allowed to stand for 4 d, purple crystalline clusters of product were obtained. This presumably THF-solvated efflorescent product was air-dried and recrystallized from nitro­methane. MS *m*/*z* = 404.117 for (*M* − Cl)^+^, calculated 404.117. Analysis C,H,N (desolvated): found %, C 49.65, H 5.84, N 13.38; calculated for C_19_H_26_Cl_2_CoN_4_: C 49.75, H 5.89, N 13.39.

**[Co(Pmhpz)Cl_2_]:** This compound was obtained by dropwise addition of crude 1-(2′-pyridyl­eth­yl)-4-methyl­homopiperazine in methanol to a warm solution of cobalt(II) chloride in methanol. After two days, the deep blue–purple solution yielded blue crystals in 55% yield. MS: observed *m*/*z* = 313.1, calculated for (*M* − Cl)^+^, 313.076. Analysis C,H,N: found %, 44.72, 5.84, 11.79; calculated for C_13_H_21_N_3_Cl_2_Co, 44.72, 6.06, 12.03.

**[Co(Ppz)Cl]ClO_4_:** The blue crystals produced were filtered off and recrystallized from aceto­nitrile. MS *m*/*z* = 390.102 (*M* − ClO_4_)^+^ = C_18_H_24_N_4_CoCl^+^, calculated 390.102. Analysis C,H,N: found %, C 44.3, H 4.78, N 11.4; calculated for C_18_H_24_N_4_CoCl_2_O_4_, C 44.1, H 4.93, N 11.4.

**[Co_2_(Pdmpz)Cl_4_]:** The blue crystals produced were filtered off and recrystallized from nitro­methane. MS *m*/*z* = 454.111, (*M* + H)^+^: calculated for C_20_H_29_Cl_2_Co_2_N_4_^+^, 454.110; *m*/*z* = 418.133, (*M* − Cl)^+^, calculated for C_20_H_28_ClCo_2_N_4_^+^, 418.133. Analysis C,H,N: found %, C 41.6, H 4.80, N 9.44; calculated for C_20_H_28_Cl_4_Co_2_N_4_: C 41.1, H 4.83, N 9.59.

## Refinement

Crystal data, data collection and structure refinement details are summarized in Table 7 ▸. X-ray diffraction data were collected on a Rigaku Oxford Diffraction Gemini diffractometer *via* ω-scans using an Atlas CCD detector using Cu *K*α radiation or a Bruker AXS D8 Quest diffractometer with a PhotonII charge-integrating pixel array detector (CPAD). Data for those structures were collected, scaled and corrected for absorption using the *CrysAlis PRO 2015* software suite program package (Rigaku OD, 2015 ▸) or *APEX4* and *SAINT* (Bruker, 2021 ▸) and *SADABS* (Krause *et al.*, 2015 ▸). Crystal structures were solved using *SHELXT* (Sheldrick, 2015*a* ▸), and refined using *SHELXL* (Sheldrick, 2015*b* ▸) and *ShelXle* (Hübschle *et al.*, 2011 ▸), with refinement by full-matrix least-squares on *F* ^2^. Further processing for the Ppz and Pmhpz complexes utilized the *OLEX* software (Dolomanov *et al.*, 2009 ▸).

The structure of Co(Phpz)Cl_2_ contains an additional 121 Å^3^ of solvent-accessible voids filled by extensively disordered nitro­methane recrystallization solvent. The residual electron density peaks are not arranged in an inter­pretable pattern. The structure factors were instead augmented *via* reverse-Fourier-transform methods using the SQUEEZE routine (van der Sluis & Spek, 1990 ▸; Spek, 2015 ▸) as implemented in *PLATON*. The resultant FAB file containing the structure-factor contribution from the electron content of the void space was used together with the original hkl file in the further refinement. (The FAB file with details of the SQUEEZE results is included in the CIF in the supporting information). The SQUEEZE procedure corrected for 69 electrons within the solvent-accessible voids, or around two nitro­methane mol­ecules. The central part of the metal complex (two of the Co-coordinated nitro­gen atoms and the C atoms bridging between them) are disordered by a pseudo-mirror operation. Additional disorder that is vaguely recognizable (largest difference peak 0.78 electrons) was ignored. The two disordered moieties were restrained to have similar geometries. *U* ^ij^ components of ADPs for disordered atoms closer to each other than 2.0 Å were restrained to be similar. Subject to these conditions, the occupancy ratio refined to 0.914 (3):0.086 (3).

For all compounds, H atoms were placed in calculated positions (C—H = 0.95–0.99 Å) and refined as riding with *U*_iso_(H) = 1.2*U*_eq_(C).

## Supplementary Material

Crystal structure: contains datablock(s) global, CoPhpzCl2_sq, ta-eab1701-c, ta-eab1607, ta-sa15-05. DOI: 10.1107/S2056989022001220/yy2006sup1.cif

Structure factors: contains datablock(s) ta-sa15-05. DOI: 10.1107/S2056989022001220/yy2006ta-sa15-05sup2.hkl

Structure factors: contains datablock(s) CoPhpzCl2_sq. DOI: 10.1107/S2056989022001220/yy2006CoPhpzCl2_sqsup3.hkl

Structure factors: contains datablock(s) ta-eab1701-c. DOI: 10.1107/S2056989022001220/yy2006ta-eab1701-csup4.hkl

Structure factors: contains datablock(s) ta-eab1607. DOI: 10.1107/S2056989022001220/yy2006ta-eab1607sup5.hkl

CCDC references: 2111922, 2111921, 2111920, 2111919

Additional supporting information:  crystallographic information; 3D view; checkCIF report

## Figures and Tables

**Figure 9:**
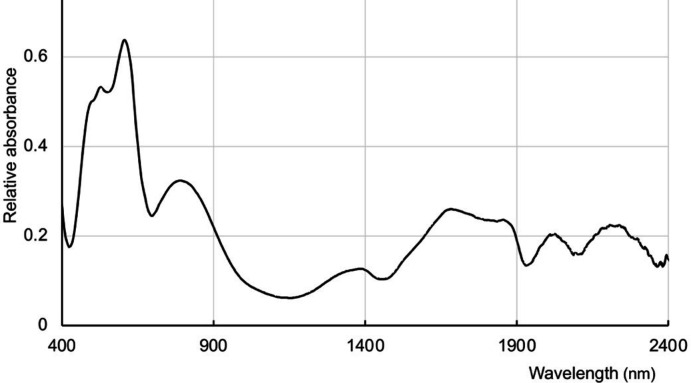
Solid-state Vis–NIR spectrum of [Co(Ppz)Cl]ClO_4_.

**Figure 11:**
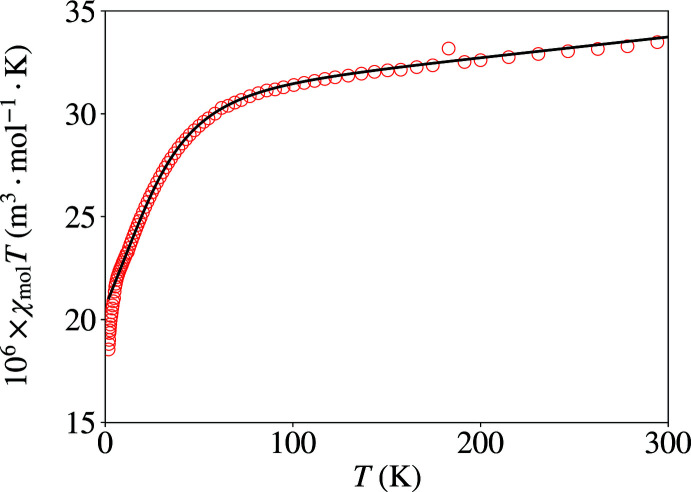
Temperature dependence of χ*T* for Co(Phpz)Cl_2_. The solid line is the fit using an exact diagonalization method, between 5 and 310 K.

**Table 1:** Selected geometric parameters (Å, °) for Co_2_(Ppz)Cl_4_ [Chem scheme1]

Co1—Cl1	2.2415 (6)	Co1—N1	2.0257 (15)
Co1—Cl2	2.2240 (6)	Co1—N2	2.0969 (15)
			
Cl2—Co1—Cl1	114.71 (2)	N1—Co1—N2	100.12 (6)
N1—Co1—Cl1	108.93 (5)	N2—Co1—Cl1	108.96 (5)
N1—Co1—Cl2	107.46 (5)	N2—Co1—Cl2	115.49 (5)

**Table 2:** Selected geometric parameters (Å, °) for Co(Pmhpz)Cl_2_ [Chem scheme1]

Co1—N2*B*	2.072 (15)	Co1—N3*B*	2.26 (3)
Co1—N2	2.0933 (15)	Co1—Cl2	2.3110 (4)
Co1—N1	2.1498 (14)	Co1—Cl1	2.3122 (4)
Co1—N3	2.228 (3)		
			
N2*B*—Co1—N1	94.8 (4)	N3—Co1—Cl2	94.48 (5)
N2—Co1—N1	94.16 (6)	N3*B*—Co1—Cl2	88.7 (6)
N2—Co1—N3	75.49 (6)	N2*B*—Co1—Cl1	114.2 (4)
N1—Co1—N3	168.81 (6)	N2—Co1—Cl1	131.72 (5)
N2*B*—Co1—N3*B*	74.9 (6)	N1—Co1—Cl1	91.92 (4)
N1—Co1—N3*B*	168.3 (4)	N3—Co1—Cl1	91.92 (5)
N2*B*—Co1—Cl2	124.4 (4)	N3*B*—Co1—Cl1	97.4 (5)
N2—Co1—Cl2	107.08 (5)	Cl2—Co1—Cl1	120.428 (18)
N1—Co1—Cl2	92.63 (4)		

**Table 3:** Selected geometric parameters (Å, °) for [Co(Ppz)Cl]ClO_4_ [Chem scheme1]

Co1*A*—N1*A*	2.057 (5)	Co1*A*—N2*A*	2.236 (5)
Co1*A*—N3*A*	2.099 (5)	Co1*A*—Cl1*A*	2.2780 (16)
Co1*A*—N4*A*	2.109 (5)		
			
N1*A*—Co1*A*—N3*A*	123.7 (2)	N4*A*—Co1*A*—N2*A*	162.6 (2)
N1*A*—Co1*A*—N4*A*	100.7 (2)	N1*A*—Co1*A*—Cl1*A*	115.11 (16)
N3*A*—Co1*A*—N4*A*	94.3 (2)	N3*A*—Co1*A*—Cl1*A*	115.81 (17)
N1*A*—Co1*A*—N2*A*	84.2 (2)	N4*A*—Co1*A*—Cl1*A*	98.25 (16)
N3*A*—Co1*A*—N2*A*	69.5 (2)	N2*A*—Co1*A*—Cl1*A*	94.62 (15)

**Table 4:** Selected geometric parameters (Å, °) for Co(Phpz)Cl_2_ [Chem scheme1]

Co1—Cl1	2.2981 (16)	Co1—N2	2.097 (4)
Co1—Cl2	2.2872 (15)	Co1—N3	2.146 (4)
Co1—N1	2.232 (5)		
			
Cl2—Co1—Cl1	118.10 (7)	N2—Co1—N1	74.86 (19)
N1—Co1—Cl1	94.21 (14)	N2—Co1—N3	93.00 (17)
N1—Co1—Cl2	92.47 (15)	N3—Co1—Cl1	93.75 (13)
N2—Co1—Cl1	108.33 (15)	N3—Co1—Cl2	92.70 (13)
N2—Co1—Cl2	132.67 (15)	N3—Co1—N1	167.11 (18)

**Table 5:** Principal absorption bands in the visible and near-IR regions

Compound	λ_max_ (nm)							
Co_2_(Ppz)Cl_4_		580	620		1040	1335	1680	
Co_2_(Pdmpz)Cl_4_		585	625		1055	1315	1680	
Co(Phpz)Cl_2_	540	565	635	783	975	1400	1664	1873
Co(Pmhpz)Cl_2_	502		635	800	990		1700	1880
[Co(Ppz)Cl]ClO_4_	540	610		810		1400	1710	1875

**Table 6:** Derived magnetism parameters for Co(Pmhpz)Cl_2_ and Co(Phpz)Cl_2_, with their estimated mean deviations

Compound	Co(Pmhpz)Cl_2_	Co(Phpz)Cl_2_
*T* window	12.5–310 K	5–310 K
*D*/*hc* (cm^−1^)	+28 (1)	+39 (1)
*g*_ave_	2.32 (2)	2.17 (2)
Δ	1.11 (6)	1.50 (10)
*a^*a*^*	0	0.00056 (21)
*b*	0.34 (5)	0.19 (2)

Note: (*a*) the *a* value for Co(Pmhpz)Cl_2_ was held at zero.

**Table 7:** Experimental details

	Co_2_(Ppz)Cl_4_	Co(Phpz)Cl_2_	[Co(Ppz)Cl]ClO_4_	Co(Pmhpz)Cl_2_
Crystal data				
Chemical formula	[Co_2_Cl_4_(C_18_H_24_N_4_)]	[CoCl_2_(C_19_H_26_N_4_)][+solvent]	[CoCl(C_18_H_24_N_4_)]ClO_4_	[CoCl_2_(C_13_H_21_N_3_)]
*M*_r_	556.07	440.27	490.24	349.16
Crystal system, space group	Monoclinic, *P*2_1_/*n*	Triclinic, *P* 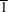	Monoclinic, *P*2_1_	Monoclinic, *P*2_1_/*n*
Temperature (K)	173	150	293	273
*a*, *b*, *c* (Å)	11.6370 (5), 7.4382 (2), 13.3104 (5)	7.2628 (3), 11.5369 (4), 12.6384 (5)	8.3952 (3), 10.9341 (4), 11.3643 (4)	10.3626 (6), 11.5871 (7), 13.7035 (7)
α, β, γ (°)	90, 104.229 (4), 90	86.9553 (19), 89.1996 (19), 89.3798 (18)	90, 92.125 (3), 90	90, 108.308 (6), 90
*V* (Å^3^)	1116.77 (7)	1057.32 (7)	1042.46 (6)	1562.12 (16)
*Z*	2	2	2	4
Radiation type	Mo *K*α	Mo *K*α	Cu *K*α	Cu *K*α
μ (mm^−1^)	1.98	1.07	9.10	11.67
Crystal size (mm)	0.32 × 0.22 × 0.11	0.23 × 0.13 × 0.09	0.18 × 0.14 × 0.12	0.42 × 0.08 × 0.06

Data collection				
Diffractometer	Agilent, Eos, Gemini	Bruker D8 Quest diffractometer with PhotonII charge-integrating pixel array detector (CPAD)	Rigaku, Oxford Diffraction Eos	Rigaku Oxford Diffraction Eos
Absorption correction	Multi-scan (*CrysAlis PRO*; Agilent, 2014 ▸)	Multi-scan (*SADABS*; Krause *et al.*, 2015 ▸)	Multi-scan (*CrysAlis PRO*; Rigaku OD, 2015 ▸)	Multi-scan (*CrysAlis PRO*; Rigaku OD, 2015 ▸)
*T*_min_, *T*_max_	0.687, 1.000	0.660, 0.747	0.378, 1.000	0.202, 1.000
No. of measured, independent and observed [*I* > 2σ(*I*)] reflections	7280, 3708, 3044	43329, 8042, 7248	6624, 3274, 2877	5711, 2957, 1805
*R*_int_	0.033	0.035	0.052	0.054
(sin θ/λ)_max_ (Å^−1^)	0.765	0.771	0.615	0.615

Refinement				
*R*[*F* ^2^ > 2σ(*F* ^2^)], *wR*(*F* ^2^), *S*	0.037, 0.095, 1.04	0.037, 0.098, 1.12	0.047, 0.116, 1.03	0.056, 0.139, 1.04
No. of reflections	3708	8042	3274	2957
No. of parameters	127	317	308	173
No. of restraints	0	298	155	0
H-atom treatment	H-atom parameters constrained	H-atom parameters constrained	H-atom parameters constrained	H-atom parameters constrained
Δρ_max_, Δρ_min_ (e Å^−3^)	0.69, −0.63	0.81, −0.35	0.77, −0.40	0.54, −0.33
Absolute structure	–	–	Classical Flack method preferred over Parsons because s.u. lower	–
Absolute structure parameter	–	–	−0.021 (7)	–

Computer programs: *CrysAlis PRO* (Agilent, 2014 ▸; Rigaku OD, 2015 ▸), *APEX4* and *SAINT* (Bruker, 2021 ▸), *SHELXT* (Sheldrick, 2015*a* ▸), *SHELXL* (Sheldrick, 2015*b* ▸), *ShelXle* (Hübschle *et al.*, 2011 ▸), and *OLEX2* (Dolomanov *et al.*, 2009 ▸).

## supplementary crystallographic information

### {µ-1,4-Bis[2-(pyridin-2-yl)ethyl]piperazine}bis[dichloridocobalt(II)] (ta-sa15-05) Crystal data

**Table:**

[Co_2_Cl_4_(C_18_H_24_N_4_)]	*F*(000) = 564
*M**_r_* = 556.07	*D*_x_ = 1.654 Mg m^−^^3^
Monoclinic, *P*2_1_/*n*	Mo *K*α radiation, λ = 0.71073 Å
*a* = 11.6370 (5) Å	Cell parameters from 2820 reflections
*b* = 7.4382 (2) Å	θ = 4.2–32.8°
*c* = 13.3104 (5) Å	µ = 1.98 mm^−^^1^
β = 104.229 (4)°	*T* = 173 K
*V* = 1116.77 (7) Å^3^	Prism, blue
*Z* = 2	0.32 × 0.22 × 0.11 mm

### {µ-1,4-Bis[2-(pyridin-2-yl)ethyl]piperazine}bis[dichloridocobalt(II)] (ta-sa15-05) Data collection

**Table:**

Agilent, Eos, Gemini diffractometer	3708 independent reflections
Radiation source: Enhance (Mo) X-ray Source	3044 reflections with *I* > 2σ(*I*)
Graphite monochromator	*R*_int_ = 0.033
Detector resolution: 16.0416 pixels mm^-1^	θ_max_ = 33.0°, θ_min_ = 3.3°
ω scans	*h* = −17→13
Absorption correction: multi-scan (CrysAlisPro; Agilent, 2014)	*k* = −9→10
*T*_min_ = 0.687, *T*_max_ = 1.000	*l* = −19→18
7280 measured reflections	

### {µ-1,4-Bis[2-(pyridin-2-yl)ethyl]piperazine}bis[dichloridocobalt(II)] (ta-sa15-05) Refinement

**Table:**

Refinement on *F*^2^	0 restraints
Least-squares matrix: full	Hydrogen site location: inferred from neighbouring sites
*R*[*F*^2^ > 2σ(*F*^2^)] = 0.037	H-atom parameters constrained
*wR*(*F*^2^) = 0.095	*w* = 1/[σ^2^(*F*_o_^2^) + (0.0428*P*)^2^] where *P* = (*F*_o_^2^ + 2*F*_c_^2^)/3
*S* = 1.04	(Δ/σ)_max_ = 0.001
3708 reflections	Δρ_max_ = 0.69 e Å^−^^3^
127 parameters	Δρ_min_ = −0.63 e Å^−^^3^

### {µ-1,4-Bis[2-(pyridin-2-yl)ethyl]piperazine}bis[dichloridocobalt(II)] (ta-sa15-05) Special details

**Table:**

Geometry. All esds (except the esd in the dihedral angle between two l.s. planes) are estimated using the full covariance matrix. The cell esds are taken into account individually in the estimation of esds in distances, angles and torsion angles; correlations between esds in cell parameters are only used when they are defined by crystal symmetry. An approximate (isotropic) treatment of cell esds is used for estimating esds involving l.s. planes.

### {µ-1,4-Bis[2-(pyridin-2-yl)ethyl]piperazine}bis[dichloridocobalt(II)] (ta-sa15-05) Fractional atomic coordinates and isotropic or equivalent isotropic displacement parameters (Å^2^)

**Table:**

	*x*	*y*	*z*	*U*_iso_*/*U*_eq_	
Co1	0.48371 (2)	0.39454 (3)	0.78675 (2)	0.01891 (8)	
Cl1	0.66943 (5)	0.29581 (7)	0.80327 (4)	0.03312 (13)	
Cl2	0.34895 (5)	0.17887 (6)	0.77648 (4)	0.02996 (13)	
N1	0.43498 (14)	0.5445 (2)	0.65615 (12)	0.0208 (3)	
N2	0.48454 (14)	0.59266 (18)	0.89900 (12)	0.0166 (3)	
C1	0.37081 (18)	0.4753 (3)	0.56620 (15)	0.0259 (4)	
H1	0.3434	0.3549	0.5654	0.031*	
C2	0.34353 (18)	0.5728 (3)	0.47548 (16)	0.0286 (4)	
H2	0.2999	0.5198	0.4127	0.034*	
C3	0.3809 (2)	0.7490 (3)	0.47777 (16)	0.0300 (4)	
H3	0.3635	0.8195	0.4164	0.036*	
C4	0.44398 (19)	0.8213 (3)	0.57021 (16)	0.0276 (4)	
H4	0.4691	0.9432	0.5731	0.033*	
C5	0.47084 (17)	0.7165 (2)	0.65901 (14)	0.0206 (4)	
C6	0.53965 (19)	0.7884 (2)	0.76234 (15)	0.0233 (4)	
H6A	0.5589	0.9165	0.7546	0.028*	
H6B	0.6152	0.7215	0.7847	0.028*	
C7	0.47009 (18)	0.7714 (2)	0.84567 (14)	0.0215 (4)	
H7A	0.4966	0.8669	0.8981	0.026*	
H7B	0.3849	0.7914	0.8133	0.026*	
C8	0.59897 (16)	0.5912 (2)	0.97969 (14)	0.0198 (4)	
H8A	0.6032	0.6989	1.0242	0.024*	
H8B	0.6655	0.5973	0.9455	0.024*	
C9	0.38779 (16)	0.5759 (2)	0.95338 (14)	0.0195 (3)	
H9A	0.3106	0.5718	0.9015	0.023*	
H9B	0.3880	0.6832	0.9974	0.023*	

### {µ-1,4-Bis[2-(pyridin-2-yl)ethyl]piperazine}bis[dichloridocobalt(II)] (ta-sa15-05) Atomic displacement parameters (Å^2^)

**Table:**

	*U*^11^	*U*^22^	*U*^33^	*U*^12^	*U*^13^	*U*^23^
Co1	0.02127 (14)	0.01542 (13)	0.01850 (14)	0.00189 (9)	0.00194 (10)	0.00053 (8)
Cl1	0.0279 (3)	0.0416 (3)	0.0308 (3)	0.0137 (2)	0.0090 (2)	0.0033 (2)
Cl2	0.0318 (3)	0.0199 (2)	0.0323 (3)	−0.00587 (19)	−0.0034 (2)	0.00083 (17)
N1	0.0204 (7)	0.0207 (7)	0.0203 (8)	0.0012 (6)	0.0029 (6)	0.0011 (6)
N2	0.0163 (7)	0.0165 (6)	0.0162 (7)	−0.0008 (6)	0.0025 (6)	0.0022 (5)
C1	0.0247 (9)	0.0278 (9)	0.0239 (10)	0.0026 (8)	0.0036 (8)	−0.0013 (7)
C2	0.0231 (10)	0.0396 (10)	0.0203 (9)	0.0040 (9)	−0.0001 (8)	−0.0013 (8)
C3	0.0279 (10)	0.0400 (11)	0.0221 (9)	0.0066 (10)	0.0061 (8)	0.0081 (8)
C4	0.0306 (11)	0.0268 (9)	0.0273 (10)	0.0014 (8)	0.0107 (9)	0.0074 (8)
C5	0.0209 (9)	0.0229 (8)	0.0192 (8)	0.0011 (7)	0.0070 (7)	0.0017 (7)
C6	0.0281 (10)	0.0199 (8)	0.0215 (9)	−0.0063 (8)	0.0053 (8)	0.0025 (7)
C7	0.0281 (10)	0.0167 (7)	0.0191 (8)	0.0019 (7)	0.0048 (7)	0.0030 (6)
C8	0.0148 (8)	0.0236 (8)	0.0196 (9)	−0.0045 (7)	0.0019 (7)	0.0036 (6)
C9	0.0156 (8)	0.0249 (8)	0.0177 (8)	0.0019 (7)	0.0035 (7)	0.0037 (6)

### {µ-1,4-Bis[2-(pyridin-2-yl)ethyl]piperazine}bis[dichloridocobalt(II)] (ta-sa15-05) Geometric parameters (Å, º)

**Table:**

Co1—Cl1	2.2415 (6)	C4—H4	0.9500
Co1—Cl2	2.2240 (6)	C4—C5	1.386 (3)
Co1—N1	2.0257 (15)	C5—C6	1.509 (3)
Co1—N2	2.0969 (15)	C6—H6A	0.9900
N1—C1	1.348 (2)	C6—H6B	0.9900
N1—C5	1.343 (2)	C6—C7	1.531 (3)
N2—C7	1.497 (2)	C7—H7A	0.9900
N2—C8	1.491 (2)	C7—H7B	0.9900
N2—C9	1.486 (2)	C8—H8A	0.9900
C1—H1	0.9500	C8—H8B	0.9900
C1—C2	1.377 (3)	C8—C9^i^	1.515 (2)
C2—H2	0.9500	C9—C8^i^	1.515 (2)
C2—C3	1.379 (3)	C9—H9A	0.9900
C3—H3	0.9500	C9—H9B	0.9900
C3—C4	1.378 (3)		
			
Cl2—Co1—Cl1	114.71 (2)	N1—C5—C4	120.55 (17)
N1—Co1—Cl1	108.93 (5)	N1—C5—C6	117.07 (16)
N1—Co1—Cl2	107.46 (5)	C4—C5—C6	122.38 (17)
N1—Co1—N2	100.12 (6)	C5—C6—H6A	109.2
N2—Co1—Cl1	108.96 (5)	C5—C6—H6B	109.2
N2—Co1—Cl2	115.49 (5)	C5—C6—C7	111.99 (16)
C1—N1—Co1	121.94 (13)	H6A—C6—H6B	107.9
C5—N1—Co1	118.73 (12)	C7—C6—H6A	109.2
C5—N1—C1	119.31 (16)	C7—C6—H6B	109.2
C7—N2—Co1	107.82 (11)	N2—C7—C6	113.52 (15)
C8—N2—Co1	110.80 (11)	N2—C7—H7A	108.9
C8—N2—C7	108.92 (14)	N2—C7—H7B	108.9
C9—N2—Co1	114.63 (11)	C6—C7—H7A	108.9
C9—N2—C7	107.17 (14)	C6—C7—H7B	108.9
C9—N2—C8	107.34 (14)	H7A—C7—H7B	107.7
N1—C1—H1	118.8	N2—C8—H8A	109.2
N1—C1—C2	122.40 (19)	N2—C8—H8B	109.2
C2—C1—H1	118.8	N2—C8—C9^i^	111.91 (14)
C1—C2—H2	120.7	H8A—C8—H8B	107.9
C1—C2—C3	118.52 (19)	C9^i^—C8—H8A	109.2
C3—C2—H2	120.7	C9^i^—C8—H8B	109.2
C2—C3—H3	120.4	N2—C9—C8^i^	112.07 (15)
C4—C3—C2	119.14 (19)	N2—C9—H9A	109.2
C4—C3—H3	120.4	N2—C9—H9B	109.2
C3—C4—H4	120.0	C8^i^—C9—H9A	109.2
C3—C4—C5	120.05 (19)	C8^i^—C9—H9B	109.2
C5—C4—H4	120.0	H9A—C9—H9B	107.9
			
Co1—N1—C1—C2	−175.85 (15)	C3—C4—C5—N1	−0.6 (3)
Co1—N1—C5—C4	177.07 (15)	C3—C4—C5—C6	180.0 (2)
Co1—N1—C5—C6	−3.5 (2)	C4—C5—C6—C7	122.0 (2)
Co1—N2—C7—C6	−39.88 (18)	C5—N1—C1—C2	2.2 (3)
Co1—N2—C8—C9^i^	−69.19 (16)	C5—C6—C7—N2	86.0 (2)
Co1—N2—C9—C8^i^	66.77 (16)	C7—N2—C8—C9^i^	172.37 (15)
N1—C1—C2—C3	−1.7 (3)	C7—N2—C9—C8^i^	−173.62 (15)
N1—C5—C6—C7	−57.4 (2)	C8—N2—C7—C6	80.41 (18)
C1—N1—C5—C4	−1.0 (3)	C8—N2—C9—C8^i^	−56.8 (2)
C1—N1—C5—C6	178.43 (17)	C9—N2—C7—C6	−163.77 (15)
C1—C2—C3—C4	0.0 (3)	C9—N2—C8—C9^i^	56.7 (2)
C2—C3—C4—C5	1.1 (3)		

Symmetry code: (i) −*x*+1, −*y*+1, −*z*+2.

### {1,4-Bis[2-(pyridin-2-yl)ethyl]-1,4-diazacycloheptane}dichloridocobalt(II) (CoPhpzCl2_sq) Crystal data

**Table:**

[CoCl_2_(C_19_H_26_N_4_)][+solvent]	*Z* = 2
*M**_r_* = 440.27	*F*(000) = 458
Triclinic, *P*1	*D*_x_ = 1.383 Mg m^−^^3^
*a* = 7.2628 (3) Å	Mo *K*α radiation, λ = 0.71073 Å
*b* = 11.5369 (4) Å	Cell parameters from 9804 reflections
*c* = 12.6384 (5) Å	θ = 3.2–33.2°
α = 86.9553 (19)°	µ = 1.07 mm^−^^1^
β = 89.1996 (19)°	*T* = 150 K
γ = 89.3798 (18)°	Fragment, blue
*V* = 1057.32 (7) Å^3^	0.23 × 0.13 × 0.09 mm

### {1,4-Bis[2-(pyridin-2-yl)ethyl]-1,4-diazacycloheptane}dichloridocobalt(II) (CoPhpzCl2_sq) Data collection

**Table:**

Bruker AXS D8 Quest diffractometer with PhotonII charge-integrating pixel array detector (CPAD)	8042 independent reflections
Radiation source: fine focus sealed tube X-ray source	7248 reflections with *I* > 2σ(*I*)
Triumph curved graphite crystal monochromator	*R*_int_ = 0.035
Detector resolution: 7.4074 pixels mm^-1^	θ_max_ = 33.2°, θ_min_ = 1.8°
ω and phi scans	*h* = −11→11
Absorption correction: multi-scan (SADABS; Krause *et al.*, 2015)	*k* = −17→17
*T*_min_ = 0.660, *T*_max_ = 0.747	*l* = −19→19
43329 measured reflections	

### {1,4-Bis[2-(pyridin-2-yl)ethyl]-1,4-diazacycloheptane}dichloridocobalt(II) (CoPhpzCl2_sq) Refinement

**Table:**

Refinement on *F*^2^	Primary atom site location: dual
Least-squares matrix: full	Secondary atom site location: difference Fourier map
*R*[*F*^2^ > 2σ(*F*^2^)] = 0.037	Hydrogen site location: inferred from neighbouring sites
*wR*(*F*^2^) = 0.098	H-atom parameters constrained
*S* = 1.12	*w* = 1/[σ^2^(*F*_o_^2^) + (0.0309*P*)^2^ + 1.165*P*] where *P* = (*F*_o_^2^ + 2*F*_c_^2^)/3
8042 reflections	(Δ/σ)_max_ = 0.002
317 parameters	Δρ_max_ = 0.81 e Å^−^^3^
298 restraints	Δρ_min_ = −0.35 e Å^−^^3^

### {1,4-Bis[2-(pyridin-2-yl)ethyl]-1,4-diazacycloheptane}dichloridocobalt(II) (CoPhpzCl2_sq) Special details

**Table:**

Geometry. All esds (except the esd in the dihedral angle between two l.s. planes) are estimated using the full covariance matrix. The cell esds are taken into account individually in the estimation of esds in distances, angles and torsion angles; correlations between esds in cell parameters are only used when they are defined by crystal symmetry. An approximate (isotropic) treatment of cell esds is used for estimating esds involving l.s. planes.
Refinement. The central part of the metal complex (two of the Co-coordinated nitrogen atoms and the C atoms bridging between them) are disordered by a pseudo-mirror operation. Addtional disorder that is vaguely recognizable (largest difference peak 0.78 electrons) was ignored. The two disordered moieties were restrained to have similar geometries. Uij components of ADPs for disordered atoms closer to each other than 2.0 Angstrom were restrained to be similar. Subject to these conditions the occupancy ratio refined to 0.914 (3) to 0.086 (3). The structure contains additional 121 Ang3 of solvent accessible voids filled by extensively disordered solvate molecules (presumably nitromethane, the solvate of crystallization). The residual electron density peaks are not arranged in an interpretable pattern. The structure factors were instead augmented via reverse Fourier transform methods using the SQUEEZE routine (P. van der Sluis & A.L. Spek (1990). Acta Cryst. A46, 194-201) as implemented in the program Platon. The resultant FAB file containing the structure factor contribution from the electron content of the void space was used in together with the original hkl file in the further refinement. (The FAB file with details of the Squeeze results is appended to this cif file). The Squeeze procedure corrected for 69 electrons within the solvent accessible voids, or around two nitromethane molecules.

### {1,4-Bis[2-(pyridin-2-yl)ethyl]-1,4-diazacycloheptane}dichloridocobalt(II) (CoPhpzCl2_sq) Fractional atomic coordinates and isotropic or equivalent isotropic displacement parameters (Å^2^)

**Table:**

	*x*	*y*	*z*	*U*_iso_*/*U*_eq_	Occ. (<1)
Co1	0.53192 (3)	0.72356 (2)	0.71546 (2)	0.01491 (5)	
Cl1	0.39576 (6)	0.90464 (3)	0.72682 (3)	0.02551 (8)	
Cl2	0.35412 (5)	0.56244 (3)	0.69120 (3)	0.02233 (8)	
N1	0.52808 (19)	0.69410 (13)	0.88487 (11)	0.0210 (2)	
N4	0.1556 (2)	0.69189 (13)	0.31011 (12)	0.0236 (3)	
C1	0.3612 (3)	0.70540 (19)	0.93121 (14)	0.0299 (4)	
H1	0.261957	0.733949	0.888753	0.036*	
C2	0.3266 (3)	0.6775 (2)	1.03773 (15)	0.0351 (4)	
H2	0.207425	0.688678	1.067724	0.042*	
C3	0.4692 (3)	0.63313 (18)	1.09915 (14)	0.0304 (4)	
H3	0.449538	0.610848	1.171842	0.036*	
C4	0.6422 (3)	0.62179 (17)	1.05229 (14)	0.0285 (3)	
H4	0.742296	0.591331	1.092985	0.034*	
C5	0.6689 (2)	0.65507 (16)	0.94559 (13)	0.0239 (3)	
N2	0.8075 (2)	0.67209 (14)	0.69839 (12)	0.0186 (3)	0.914 (3)
N3	0.5951 (3)	0.75652 (17)	0.5435 (2)	0.0161 (3)	0.914 (3)
C6	0.8595 (4)	0.6501 (3)	0.8962 (2)	0.0278 (6)	0.914 (3)
H6A	0.907241	0.730202	0.888560	0.033*	0.914 (3)
H6B	0.940719	0.605601	0.946249	0.033*	0.914 (3)
C7	0.8759 (3)	0.59720 (18)	0.79009 (16)	0.0256 (4)	0.914 (3)
H7A	1.007023	0.577766	0.776770	0.031*	0.914 (3)
H7B	0.806603	0.523656	0.793394	0.031*	0.914 (3)
C8	0.8212 (3)	0.60193 (16)	0.60292 (16)	0.0220 (3)	0.914 (3)
H8A	0.787981	0.520602	0.622862	0.026*	0.914 (3)
H8B	0.950000	0.602210	0.576264	0.026*	0.914 (3)
C9	0.6934 (3)	0.64957 (17)	0.51413 (18)	0.0193 (4)	0.914 (3)
H9A	0.767374	0.666467	0.448841	0.023*	0.914 (3)
H9B	0.602312	0.589692	0.498837	0.023*	0.914 (3)
C10	0.7153 (3)	0.85978 (18)	0.5286 (2)	0.0203 (4)	0.914 (3)
H10A	0.658570	0.925301	0.565159	0.024*	0.914 (3)
H10B	0.724466	0.882734	0.452098	0.024*	0.914 (3)
C11	0.9089 (3)	0.83628 (17)	0.57172 (17)	0.0244 (4)	0.914 (3)
H11A	0.975384	0.785361	0.523062	0.029*	0.914 (3)
H11B	0.975306	0.910862	0.570387	0.029*	0.914 (3)
C12	0.9187 (2)	0.78025 (17)	0.68365 (16)	0.0239 (3)	0.914 (3)
H12A	1.048899	0.761401	0.700064	0.029*	0.914 (3)
H12B	0.874315	0.836870	0.734617	0.029*	0.914 (3)
N2B	0.817 (2)	0.7263 (13)	0.7020 (10)	0.0189 (19)	0.086 (3)
N3B	0.592 (3)	0.7352 (19)	0.539 (2)	0.018 (3)	0.086 (3)
C6B	0.857 (3)	0.674 (4)	0.897 (2)	0.027 (3)	0.086 (3)
H6C	0.936090	0.698669	0.954391	0.032*	0.086 (3)
H6D	0.903055	0.595936	0.878877	0.032*	0.086 (3)
C7B	0.903 (3)	0.7523 (18)	0.8028 (13)	0.028 (2)	0.086 (3)
H7C	0.867783	0.832473	0.819544	0.033*	0.086 (3)
H7D	1.038393	0.750743	0.791949	0.033*	0.086 (3)
C8B	0.869 (3)	0.8190 (15)	0.6206 (14)	0.022 (2)	0.086 (3)
H8C	0.992689	0.800892	0.590990	0.027*	0.086 (3)
H8D	0.876643	0.894227	0.654440	0.027*	0.086 (3)
C9B	0.727 (4)	0.829 (2)	0.529 (2)	0.021 (3)	0.086 (3)
H9C	0.662432	0.905059	0.530854	0.026*	0.086 (3)
H9D	0.793098	0.826209	0.460360	0.026*	0.086 (3)
C10B	0.663 (3)	0.6218 (19)	0.508 (2)	0.020 (2)	0.086 (3)
H10C	0.662423	0.619746	0.429500	0.025*	0.086 (3)
H10D	0.580853	0.559595	0.537089	0.025*	0.086 (3)
C11B	0.860 (3)	0.5997 (18)	0.5482 (14)	0.027 (2)	0.086 (3)
H11C	0.900632	0.520830	0.530291	0.033*	0.086 (3)
H11D	0.944096	0.656140	0.511618	0.033*	0.086 (3)
C12B	0.873 (3)	0.6104 (15)	0.6666 (13)	0.027 (2)	0.086 (3)
H12C	0.793950	0.550760	0.702889	0.032*	0.086 (3)
H12D	1.001477	0.594237	0.688289	0.032*	0.086 (3)
C13	0.4219 (2)	0.77299 (15)	0.48334 (12)	0.0197 (3)	
H13A	0.362629	0.846289	0.503844	0.024*	
H13B	0.337699	0.708954	0.505272	0.024*	
C14	0.4423 (2)	0.77715 (16)	0.36172 (13)	0.0232 (3)	
H14A	0.519445	0.843907	0.337483	0.028*	
H14B	0.503654	0.705223	0.339373	0.028*	
C15	0.2548 (2)	0.78897 (14)	0.31256 (12)	0.0202 (3)	
C16	−0.0139 (2)	0.70104 (17)	0.26944 (14)	0.0257 (3)	
H16	−0.084107	0.632313	0.266619	0.031*	
C17	−0.0923 (3)	0.80415 (19)	0.23144 (16)	0.0303 (4)	
H17	−0.212622	0.806205	0.202799	0.036*	
C18	0.0093 (3)	0.90462 (19)	0.23624 (19)	0.0369 (4)	
H18	−0.041362	0.977641	0.212811	0.044*	
C19	0.1866 (3)	0.89658 (17)	0.27597 (17)	0.0310 (4)	
H19	0.260430	0.963874	0.278109	0.037*	

### {1,4-Bis[2-(pyridin-2-yl)ethyl]-1,4-diazacycloheptane}dichloridocobalt(II) (CoPhpzCl2_sq) Atomic displacement parameters (Å^2^)

**Table:**

	*U*^11^	*U*^22^	*U*^33^	*U*^12^	*U*^13^	*U*^23^
Co1	0.01236 (9)	0.01649 (9)	0.01594 (9)	0.00004 (6)	−0.00004 (6)	−0.00142 (7)
Cl1	0.02846 (19)	0.02000 (16)	0.02813 (19)	0.00544 (14)	0.00301 (14)	−0.00377 (14)
Cl2	0.02063 (16)	0.02148 (16)	0.02497 (17)	−0.00612 (13)	0.00151 (13)	−0.00138 (13)
N1	0.0191 (6)	0.0269 (7)	0.0171 (6)	0.0007 (5)	−0.0007 (4)	−0.0013 (5)
N4	0.0251 (7)	0.0239 (6)	0.0218 (6)	−0.0030 (5)	0.0003 (5)	0.0003 (5)
C1	0.0237 (8)	0.0462 (11)	0.0193 (7)	0.0058 (7)	0.0023 (6)	0.0004 (7)
C2	0.0323 (10)	0.0518 (12)	0.0207 (8)	0.0046 (8)	0.0066 (7)	−0.0012 (8)
C3	0.0410 (10)	0.0349 (9)	0.0153 (7)	−0.0006 (8)	0.0008 (6)	−0.0020 (6)
C4	0.0342 (9)	0.0331 (9)	0.0182 (7)	0.0033 (7)	−0.0055 (6)	−0.0004 (6)
C5	0.0251 (7)	0.0276 (8)	0.0191 (7)	0.0026 (6)	−0.0038 (6)	−0.0011 (6)
N2	0.0141 (6)	0.0213 (7)	0.0202 (6)	0.0008 (5)	0.0010 (5)	0.0009 (5)
N3	0.0150 (6)	0.0164 (8)	0.0169 (7)	−0.0002 (6)	0.0001 (5)	−0.0015 (6)
C6	0.0209 (9)	0.0345 (16)	0.0278 (10)	0.0016 (8)	−0.0061 (7)	0.0021 (9)
C7	0.0198 (8)	0.0299 (9)	0.0265 (8)	0.0048 (6)	−0.0005 (6)	0.0042 (7)
C8	0.0198 (7)	0.0214 (7)	0.0246 (8)	0.0035 (6)	0.0036 (6)	−0.0006 (6)
C9	0.0200 (8)	0.0190 (8)	0.0192 (7)	−0.0003 (6)	0.0034 (6)	−0.0039 (7)
C10	0.0220 (8)	0.0184 (9)	0.0203 (7)	−0.0041 (8)	0.0004 (6)	0.0015 (7)
C11	0.0194 (8)	0.0259 (8)	0.0277 (9)	−0.0076 (6)	0.0023 (6)	0.0014 (7)
C12	0.0175 (7)	0.0276 (8)	0.0268 (8)	−0.0051 (6)	−0.0034 (6)	−0.0006 (7)
N2B	0.015 (3)	0.019 (4)	0.022 (3)	−0.008 (3)	−0.002 (3)	0.002 (3)
N3B	0.018 (4)	0.020 (5)	0.017 (4)	−0.006 (4)	0.001 (4)	0.001 (4)
C6B	0.020 (5)	0.035 (6)	0.025 (5)	0.003 (5)	−0.008 (5)	0.001 (5)
C7B	0.019 (4)	0.036 (4)	0.028 (4)	−0.002 (4)	−0.004 (4)	0.003 (4)
C8B	0.020 (4)	0.023 (4)	0.023 (4)	−0.008 (4)	0.002 (4)	0.004 (4)
C9B	0.023 (4)	0.022 (5)	0.019 (4)	0.001 (4)	0.003 (4)	0.005 (4)
C10B	0.020 (4)	0.020 (5)	0.021 (4)	0.000 (4)	0.002 (4)	−0.003 (4)
C11B	0.027 (5)	0.028 (5)	0.026 (5)	0.001 (4)	0.006 (4)	0.001 (4)
C12B	0.022 (4)	0.030 (4)	0.028 (4)	0.000 (4)	0.006 (4)	0.002 (4)
C13	0.0177 (6)	0.0252 (7)	0.0161 (6)	0.0001 (5)	−0.0006 (5)	−0.0006 (5)
C14	0.0203 (7)	0.0324 (8)	0.0169 (6)	−0.0006 (6)	−0.0014 (5)	−0.0014 (6)
C15	0.0223 (7)	0.0241 (7)	0.0145 (6)	−0.0007 (5)	−0.0006 (5)	−0.0023 (5)
C16	0.0237 (7)	0.0312 (8)	0.0225 (7)	−0.0067 (6)	0.0024 (6)	−0.0030 (6)
C17	0.0225 (8)	0.0384 (10)	0.0305 (9)	0.0002 (7)	−0.0042 (6)	−0.0042 (7)
C18	0.0353 (10)	0.0291 (9)	0.0463 (12)	0.0048 (8)	−0.0127 (9)	−0.0005 (8)
C19	0.0330 (9)	0.0225 (8)	0.0378 (10)	−0.0019 (7)	−0.0104 (8)	−0.0015 (7)

### {1,4-Bis[2-(pyridin-2-yl)ethyl]-1,4-diazacycloheptane}dichloridocobalt(II) (CoPhpzCl2_sq) Geometric parameters (Å, º)

**Table:**

Co1—N2B	2.072 (15)	C11—H11B	0.9900
Co1—N2	2.0933 (15)	C12—H12A	0.9900
Co1—N1	2.1498 (14)	C12—H12B	0.9900
Co1—N3	2.228 (3)	N2B—C7B	1.475 (15)
Co1—N3B	2.26 (3)	N2B—C12B	1.485 (16)
Co1—Cl2	2.3110 (4)	N2B—C8B	1.493 (15)
Co1—Cl1	2.3122 (4)	N3B—C9B	1.471 (18)
N1—C5	1.347 (2)	N3B—C10B	1.473 (18)
N1—C1	1.347 (2)	N3B—C13	1.481 (15)
N4—C15	1.341 (2)	C6B—C7B	1.489 (18)
N4—C16	1.341 (2)	C6B—H6C	0.9900
C1—C2	1.388 (3)	C6B—H6D	0.9900
C1—H1	0.9500	C7B—H7C	0.9900
C2—C3	1.381 (3)	C7B—H7D	0.9900
C2—H2	0.9500	C8B—C9B	1.557 (17)
C3—C4	1.389 (3)	C8B—H8C	0.9900
C3—H3	0.9500	C8B—H8D	0.9900
C4—C5	1.394 (2)	C9B—H9C	0.9900
C4—H4	0.9500	C9B—H9D	0.9900
C5—C6B	1.508 (18)	C10B—C11B	1.542 (17)
C5—C6	1.512 (3)	C10B—H10C	0.9900
N2—C8	1.490 (2)	C10B—H10D	0.9900
N2—C7	1.496 (2)	C11B—C12B	1.512 (17)
N2—C12	1.496 (2)	C11B—H11C	0.9900
N3—C9	1.481 (3)	C11B—H11D	0.9900
N3—C13	1.484 (2)	C12B—H12C	0.9900
N3—C10	1.487 (3)	C12B—H12D	0.9900
C6—C7	1.505 (4)	C13—C14	1.540 (2)
C6—H6A	0.9900	C13—H13A	0.9900
C6—H6B	0.9900	C13—H13B	0.9900
C7—H7A	0.9900	C14—C15	1.506 (2)
C7—H7B	0.9900	C14—H14A	0.9900
C8—C9	1.541 (3)	C14—H14B	0.9900
C8—H8A	0.9900	C15—C19	1.390 (2)
C8—H8B	0.9900	C16—C17	1.379 (3)
C9—H9A	0.9900	C16—H16	0.9500
C9—H9B	0.9900	C17—C18	1.385 (3)
C10—C11	1.532 (3)	C17—H17	0.9500
C10—H10A	0.9900	C18—C19	1.389 (3)
C10—H10B	0.9900	C18—H18	0.9500
C11—C12	1.526 (3)	C19—H19	0.9500
C11—H11A	0.9900		
			
N2B—Co1—N1	94.8 (4)	N2—C12—H12A	108.9
N2—Co1—N1	94.16 (6)	C11—C12—H12A	108.9
N2—Co1—N3	75.49 (6)	N2—C12—H12B	108.9
N1—Co1—N3	168.81 (6)	C11—C12—H12B	108.9
N2B—Co1—N3B	74.9 (6)	H12A—C12—H12B	107.7
N1—Co1—N3B	168.3 (4)	C7B—N2B—C12B	111.9 (14)
N2B—Co1—Cl2	124.4 (4)	C7B—N2B—C8B	108.1 (13)
N2—Co1—Cl2	107.08 (5)	C12B—N2B—C8B	110.4 (13)
N1—Co1—Cl2	92.63 (4)	C7B—N2B—Co1	111.8 (11)
N3—Co1—Cl2	94.48 (5)	C12B—N2B—Co1	106.0 (11)
N3B—Co1—Cl2	88.7 (6)	C8B—N2B—Co1	108.6 (10)
N2B—Co1—Cl1	114.2 (4)	C9B—N3B—C10B	114 (2)
N2—Co1—Cl1	131.72 (5)	C9B—N3B—C13	109.1 (17)
N1—Co1—Cl1	91.92 (4)	C10B—N3B—C13	113.2 (16)
N3—Co1—Cl1	91.92 (5)	C9B—N3B—Co1	102.1 (15)
N3B—Co1—Cl1	97.4 (5)	C10B—N3B—Co1	108.6 (17)
Cl2—Co1—Cl1	120.428 (18)	C13—N3B—Co1	108.7 (16)
C5—N1—C1	118.13 (15)	C7B—C6B—C5	126 (2)
C5—N1—Co1	126.68 (12)	C7B—C6B—H6C	105.7
C1—N1—Co1	114.83 (11)	C5—C6B—H6C	105.7
C15—N4—C16	117.68 (16)	C7B—C6B—H6D	105.7
N1—C1—C2	123.43 (18)	C5—C6B—H6D	105.7
N1—C1—H1	118.3	H6C—C6B—H6D	106.2
C2—C1—H1	118.3	N2B—C7B—C6B	117 (2)
C3—C2—C1	118.49 (18)	N2B—C7B—H7C	108.1
C3—C2—H2	120.8	C6B—C7B—H7C	108.1
C1—C2—H2	120.8	N2B—C7B—H7D	108.1
C2—C3—C4	118.52 (17)	C6B—C7B—H7D	108.1
C2—C3—H3	120.7	H7C—C7B—H7D	107.3
C4—C3—H3	120.7	N2B—C8B—C9B	111.2 (13)
C3—C4—C5	120.04 (17)	N2B—C8B—H8C	109.4
C3—C4—H4	120.0	C9B—C8B—H8C	109.4
C5—C4—H4	120.0	N2B—C8B—H8D	109.4
N1—C5—C4	121.30 (17)	C9B—C8B—H8D	109.4
N1—C5—C6B	114.8 (10)	H8C—C8B—H8D	108.0
C4—C5—C6B	122.7 (11)	N3B—C9B—C8B	111.3 (15)
N1—C5—C6	118.58 (18)	N3B—C9B—H9C	109.4
C4—C5—C6	120.10 (18)	C8B—C9B—H9C	109.4
C8—N2—C7	107.13 (15)	N3B—C9B—H9D	109.4
C8—N2—C12	110.95 (14)	C8B—C9B—H9D	109.4
C7—N2—C12	110.86 (15)	H9C—C9B—H9D	108.0
C8—N2—Co1	107.43 (11)	N3B—C10B—C11B	110.9 (15)
C7—N2—Co1	113.33 (11)	N3B—C10B—H10C	109.5
C12—N2—Co1	107.12 (11)	C11B—C10B—H10C	109.5
C9—N3—C13	110.98 (17)	N3B—C10B—H10D	109.5
C9—N3—C10	111.21 (17)	C11B—C10B—H10D	109.5
C13—N3—C10	111.21 (18)	H10C—C10B—H10D	108.1
C9—N3—Co1	103.56 (15)	C12B—C11B—C10B	112.3 (16)
C13—N3—Co1	110.15 (16)	C12B—C11B—H11C	109.2
C10—N3—Co1	109.47 (14)	C10B—C11B—H11C	109.2
C7—C6—C5	116.7 (2)	C12B—C11B—H11D	109.2
C7—C6—H6A	108.1	C10B—C11B—H11D	109.2
C5—C6—H6A	108.1	H11C—C11B—H11D	107.9
C7—C6—H6B	108.1	N2B—C12B—C11B	113.6 (14)
C5—C6—H6B	108.1	N2B—C12B—H12C	108.8
H6A—C6—H6B	107.3	C11B—C12B—H12C	108.8
N2—C7—C6	115.13 (18)	N2B—C12B—H12D	108.8
N2—C7—H7A	108.5	C11B—C12B—H12D	108.8
C6—C7—H7A	108.5	H12C—C12B—H12D	107.7
N2—C7—H7B	108.5	N3B—C13—C14	113.5 (13)
C6—C7—H7B	108.5	N3—C13—C14	115.92 (16)
H7A—C7—H7B	107.5	N3—C13—H13A	108.3
N2—C8—C9	111.81 (15)	C14—C13—H13A	108.3
N2—C8—H8A	109.3	N3—C13—H13B	108.3
C9—C8—H8A	109.3	C14—C13—H13B	108.3
N2—C8—H8B	109.3	H13A—C13—H13B	107.4
C9—C8—H8B	109.3	C15—C14—C13	109.50 (13)
H8A—C8—H8B	107.9	C15—C14—H14A	109.8
N3—C9—C8	111.89 (17)	C13—C14—H14A	109.8
N3—C9—H9A	109.2	C15—C14—H14B	109.8
C8—C9—H9A	109.2	C13—C14—H14B	109.8
N3—C9—H9B	109.2	H14A—C14—H14B	108.2
C8—C9—H9B	109.2	N4—C15—C19	122.17 (16)
H9A—C9—H9B	107.9	N4—C15—C14	116.69 (15)
N3—C10—C11	112.12 (17)	C19—C15—C14	121.08 (16)
N3—C10—H10A	109.2	N4—C16—C17	123.99 (17)
C11—C10—H10A	109.2	N4—C16—H16	118.0
N3—C10—H10B	109.2	C17—C16—H16	118.0
C11—C10—H10B	109.2	C16—C17—C18	118.11 (18)
H10A—C10—H10B	107.9	C16—C17—H17	120.9
C12—C11—C10	116.03 (17)	C18—C17—H17	120.9
C12—C11—H11A	108.3	C17—C18—C19	118.75 (19)
C10—C11—H11A	108.3	C17—C18—H18	120.6
C12—C11—H11B	108.3	C19—C18—H18	120.6
C10—C11—H11B	108.3	C18—C19—C15	119.27 (18)
H11A—C11—H11B	107.4	C18—C19—H19	120.4
N2—C12—C11	113.26 (15)	C15—C19—H19	120.4
			
C5—N1—C1—C2	−0.9 (3)	C12B—N2B—C7B—C6B	−62 (2)
Co1—N1—C1—C2	172.64 (18)	C8B—N2B—C7B—C6B	176 (2)
N1—C1—C2—C3	−1.7 (4)	Co1—N2B—C7B—C6B	57 (2)
C1—C2—C3—C4	2.0 (3)	C5—C6B—C7B—N2B	−62 (4)
C2—C3—C4—C5	0.1 (3)	C7B—N2B—C8B—C9B	−156.5 (19)
C1—N1—C5—C4	3.0 (3)	C12B—N2B—C8B—C9B	81 (2)
Co1—N1—C5—C4	−169.60 (14)	Co1—N2B—C8B—C9B	−35 (2)
C1—N1—C5—C6B	−165 (2)	C10B—N3B—C9B—C8B	−76 (3)
Co1—N1—C5—C6B	23 (2)	C13—N3B—C9B—C8B	156 (2)
C1—N1—C5—C6	−175.7 (2)	Co1—N3B—C9B—C8B	41 (2)
Co1—N1—C5—C6	11.6 (3)	N2B—C8B—C9B—N3B	−8 (3)
C3—C4—C5—N1	−2.7 (3)	C9B—N3B—C10B—C11B	41 (3)
C3—C4—C5—C6B	164 (2)	C13—N3B—C10B—C11B	167 (2)
C3—C4—C5—C6	176.0 (2)	Co1—N3B—C10B—C11B	−72 (2)
N1—C5—C6—C7	−46.8 (3)	N3B—C10B—C11B—C12B	55 (3)
C4—C5—C6—C7	134.4 (2)	C7B—N2B—C12B—C11B	−154.9 (16)
C8—N2—C7—C6	−175.57 (18)	C8B—N2B—C12B—C11B	−34 (2)
C12—N2—C7—C6	63.2 (2)	Co1—N2B—C12B—C11B	83.0 (16)
Co1—N2—C7—C6	−57.3 (2)	C10B—C11B—C12B—N2B	−59 (2)
C5—C6—C7—N2	74.9 (3)	C9B—N3B—C13—C14	73 (2)
C7—N2—C8—C9	159.07 (16)	C10B—N3B—C13—C14	−56 (2)
C12—N2—C8—C9	−79.80 (18)	Co1—N3B—C13—C14	−176.5 (5)
Co1—N2—C8—C9	36.99 (17)	C9—N3—C13—C14	−56.5 (2)
C13—N3—C9—C8	−155.72 (19)	C10—N3—C13—C14	67.8 (2)
C10—N3—C9—C8	79.9 (2)	Co1—N3—C13—C14	−170.61 (12)
Co1—N3—C9—C8	−37.55 (18)	N3B—C13—C14—C15	167.0 (10)
N2—C8—C9—N3	2.7 (2)	N3—C13—C14—C15	177.57 (15)
C9—N3—C10—C11	−44.7 (3)	C16—N4—C15—C19	0.8 (3)
C13—N3—C10—C11	−168.9 (2)	C16—N4—C15—C14	178.14 (15)
Co1—N3—C10—C11	69.10 (19)	C13—C14—C15—N4	−79.46 (18)
N3—C10—C11—C12	−48.9 (3)	C13—C14—C15—C19	97.9 (2)
C8—N2—C12—C11	38.9 (2)	C15—N4—C16—C17	−0.9 (3)
C7—N2—C12—C11	157.83 (16)	N4—C16—C17—C18	−0.5 (3)
Co1—N2—C12—C11	−78.06 (16)	C16—C17—C18—C19	1.9 (3)
C10—C11—C12—N2	52.5 (2)	C17—C18—C19—C15	−1.9 (3)
N1—C5—C6B—C7B	15 (4)	N4—C15—C19—C18	0.5 (3)
C4—C5—C6B—C7B	−153 (3)	C14—C15—C19—C18	−176.65 (19)

### {1,4-Bis[2-(pyridin-2-yl)ethyl]piperazine}chloridocobalt(II) perchlorate (ta-eab1701-c) Crystal data

**Table:**

[CoCl(C_18_H_24_N_4_)]ClO_4_	*F*(000) = 506
*M**_r_* = 490.24	*D*_x_ = 1.562 Mg m^−^^3^
Monoclinic, *P*2_1_	Cu *K*α radiation, λ = 1.54184 Å
*a* = 8.3952 (3) Å	Cell parameters from 2470 reflections
*b* = 10.9341 (4) Å	θ = 3.9–71.3°
*c* = 11.3643 (4) Å	µ = 9.10 mm^−^^1^
β = 92.125 (3)°	*T* = 293 K
*V* = 1042.46 (6) Å^3^	Prism, violet
*Z* = 2	0.18 × 0.14 × 0.12 mm

### {1,4-Bis[2-(pyridin-2-yl)ethyl]piperazine}chloridocobalt(II) perchlorate (ta-eab1701-c) Data collection

**Table:**

Rigaku, Oxford diffraction diffractometer	3274 independent reflections
Radiation source: fine-focus sealed X-ray tube, Enhance (Cu) X-ray Source	2877 reflections with *I* > 2σ(*I*)
Graphite monochromator	*R*_int_ = 0.052
Detector resolution: 16.0416 pixels mm^-1^	θ_max_ = 71.5°, θ_min_ = 3.9°
ω scans	*h* = −9→10
Absorption correction: multi-scan (CrysAlisPro; Rigaku OD, 2015)	*k* = −13→10
*T*_min_ = 0.378, *T*_max_ = 1.000	*l* = −12→13
6624 measured reflections	

### {1,4-Bis[2-(pyridin-2-yl)ethyl]piperazine}chloridocobalt(II) perchlorate (ta-eab1701-c) Refinement

**Table:**

Refinement on *F*^2^	Secondary atom site location: difference Fourier map
Least-squares matrix: full	Hydrogen site location: inferred from neighbouring sites
*R*[*F*^2^ > 2σ(*F*^2^)] = 0.047	H-atom parameters constrained
*wR*(*F*^2^) = 0.116	*w* = 1/[σ^2^(*F*_o_^2^) + (0.0576*P*)^2^] where *P* = (*F*_o_^2^ + 2*F*_c_^2^)/3
*S* = 1.03	(Δ/σ)_max_ = 0.002
3274 reflections	Δρ_max_ = 0.77 e Å^−^^3^
308 parameters	Δρ_min_ = −0.40 e Å^−^^3^
155 restraints	Absolute structure: Classical Flack method preferred over Parsons because s.u. lower
Primary atom site location: dual	Absolute structure parameter: −0.021 (7)

### {1,4-Bis[2-(pyridin-2-yl)ethyl]piperazine}chloridocobalt(II) perchlorate (ta-eab1701-c) Special details

**Table:**

Geometry. All esds (except the esd in the dihedral angle between two l.s. planes) are estimated using the full covariance matrix. The cell esds are taken into account individually in the estimation of esds in distances, angles and torsion angles; correlations between esds in cell parameters are only used when they are defined by crystal symmetry. An approximate (isotropic) treatment of cell esds is used for estimating esds involving l.s. planes.
Refinement. The perchlorate ion was refined as disordered by a slight rotation. The two disordered moieties were restrained to have similar geometries. Uij components of ADPs for disordered atoms closer to each other than 2.0 Angstrom were restrained to be similar. Subject to these conditions the occupancy ratio refined to 0.540 (19) to 0.460 (19).

### {1,4-Bis[2-(pyridin-2-yl)ethyl]piperazine}chloridocobalt(II) perchlorate (ta-eab1701-c) Fractional atomic coordinates and isotropic or equivalent isotropic displacement parameters (Å^2^)

**Table:**

	*x*	*y*	*z*	*U*_iso_*/*U*_eq_	Occ. (<1)
Co1A	0.55461 (10)	0.55297 (7)	0.83474 (7)	0.0299 (2)	
Cl1A	0.4381 (2)	0.5403 (2)	1.01221 (12)	0.0572 (5)	
N1A	0.6426 (6)	0.3908 (5)	0.7724 (4)	0.0328 (11)	
N2A	0.3341 (5)	0.5009 (5)	0.7303 (5)	0.0378 (12)	
N3A	0.4779 (6)	0.6982 (5)	0.7259 (5)	0.0369 (12)	
N4A	0.7676 (6)	0.6407 (5)	0.8916 (5)	0.0362 (11)	
C1A	0.7876 (7)	0.3864 (6)	0.7261 (6)	0.0363 (13)	
H1A	0.850157	0.456584	0.728812	0.044*	
C2A	0.8478 (8)	0.2833 (7)	0.6751 (6)	0.0462 (17)	
H2A	0.948525	0.284273	0.643694	0.055*	
C3A	0.7578 (9)	0.1795 (7)	0.6710 (6)	0.0476 (17)	
H3A	0.797059	0.108202	0.638103	0.057*	
C4A	0.6069 (9)	0.1819 (6)	0.7166 (6)	0.0418 (15)	
H4A	0.543737	0.112018	0.714020	0.050*	
C5A	0.5508 (8)	0.2878 (6)	0.7657 (6)	0.0335 (14)	
C6A	0.3877 (8)	0.2942 (7)	0.8161 (6)	0.0437 (15)	
H6AA	0.398062	0.323980	0.896421	0.052*	
H6AB	0.343464	0.212291	0.818602	0.052*	
C7A	0.2709 (8)	0.3772 (8)	0.7460 (7)	0.0485 (17)	
H7AA	0.247778	0.341021	0.669318	0.058*	
H7AB	0.171676	0.382148	0.786883	0.058*	
C8A	0.2215 (8)	0.5989 (8)	0.7598 (7)	0.0505 (17)	
H8AA	0.175124	0.581761	0.834998	0.061*	
H8AB	0.135983	0.603416	0.700176	0.061*	
C9A	0.3123 (9)	0.7207 (7)	0.7661 (8)	0.053 (2)	
H9AA	0.259024	0.780907	0.715770	0.064*	
H9AB	0.316132	0.751531	0.846192	0.064*	
C10A	0.3831 (7)	0.5229 (7)	0.6094 (5)	0.0427 (16)	
H10A	0.290829	0.520504	0.555489	0.051*	
H10B	0.457253	0.460105	0.586342	0.051*	
C11A	0.4623 (9)	0.6483 (8)	0.6049 (6)	0.0475 (17)	
H11A	0.566723	0.641025	0.571727	0.057*	
H11B	0.398320	0.703001	0.555292	0.057*	
C12A	0.5736 (9)	0.8108 (7)	0.7317 (7)	0.0449 (17)	
H12A	0.553124	0.853475	0.804460	0.054*	
H12B	0.541105	0.863798	0.666769	0.054*	
C13A	0.7532 (9)	0.7846 (7)	0.7263 (6)	0.0454 (16)	
H13A	0.769664	0.723368	0.666230	0.054*	
H13B	0.806884	0.858794	0.702779	0.054*	
C14A	0.8280 (7)	0.7406 (6)	0.8409 (5)	0.0347 (13)	
C15A	0.9611 (8)	0.8001 (7)	0.8921 (7)	0.0449 (16)	
H15A	1.002814	0.869074	0.856521	0.054*	
C16A	1.0294 (8)	0.7562 (8)	0.9948 (7)	0.0511 (19)	
H16A	1.117969	0.795338	1.028881	0.061*	
C17A	0.9692 (8)	0.6564 (8)	1.0469 (6)	0.0496 (18)	
H17A	1.015624	0.625491	1.116260	0.060*	
C18A	0.8361 (8)	0.6013 (7)	0.9942 (6)	0.0416 (14)	
H18A	0.791831	0.534196	1.031197	0.050*	
Cl1B	0.8515 (11)	0.5211 (9)	0.4162 (9)	0.041 (2)	0.540 (19)
O1B	0.9721 (18)	0.4337 (16)	0.4341 (17)	0.081 (4)	0.540 (19)
O2B	0.841 (2)	0.551 (2)	0.2956 (13)	0.082 (4)	0.540 (19)
O3B	0.698 (2)	0.477 (2)	0.450 (3)	0.064 (5)	0.540 (19)
O4B	0.891 (2)	0.6189 (19)	0.4913 (17)	0.098 (5)	0.540 (19)
Cl1C	0.8480 (15)	0.5254 (12)	0.4181 (12)	0.050 (3)	0.460 (19)
O1C	0.9811 (19)	0.495 (2)	0.4894 (18)	0.089 (5)	0.460 (19)
O2C	0.891 (3)	0.510 (2)	0.3011 (16)	0.078 (5)	0.460 (19)
O3C	0.721 (2)	0.446 (2)	0.447 (3)	0.056 (5)	0.460 (19)
O4C	0.822 (2)	0.6509 (13)	0.4417 (18)	0.069 (4)	0.460 (19)

### {1,4-Bis[2-(pyridin-2-yl)ethyl]piperazine}chloridocobalt(II) perchlorate (ta-eab1701-c) Atomic displacement parameters (Å^2^)

**Table:**

	*U*^11^	*U*^22^	*U*^33^	*U*^12^	*U*^13^	*U*^23^
Co1A	0.0308 (4)	0.0292 (5)	0.0295 (4)	−0.0003 (4)	−0.0008 (3)	0.0007 (4)
Cl1A	0.0685 (9)	0.0720 (12)	0.0319 (6)	−0.0196 (11)	0.0101 (6)	−0.0027 (9)
N1A	0.030 (2)	0.034 (3)	0.033 (2)	0.000 (2)	−0.0033 (19)	−0.001 (2)
N2A	0.023 (2)	0.048 (3)	0.042 (3)	0.001 (2)	−0.0016 (18)	−0.001 (2)
N3A	0.037 (3)	0.037 (3)	0.037 (3)	0.008 (2)	−0.002 (2)	0.002 (2)
N4A	0.037 (3)	0.034 (3)	0.037 (3)	−0.006 (2)	−0.001 (2)	−0.002 (2)
C1A	0.027 (3)	0.039 (4)	0.043 (3)	0.003 (3)	−0.002 (2)	0.001 (3)
C2A	0.032 (3)	0.060 (5)	0.046 (4)	0.010 (3)	−0.004 (3)	−0.005 (3)
C3A	0.052 (4)	0.049 (4)	0.041 (3)	0.014 (3)	−0.007 (3)	−0.011 (3)
C4A	0.052 (4)	0.030 (3)	0.043 (3)	−0.001 (3)	−0.004 (3)	−0.005 (3)
C5A	0.037 (3)	0.029 (3)	0.035 (3)	0.001 (3)	0.001 (2)	0.009 (2)
C6A	0.043 (3)	0.037 (4)	0.051 (4)	−0.014 (3)	0.008 (3)	0.002 (3)
C7A	0.032 (3)	0.054 (5)	0.059 (4)	−0.011 (3)	0.001 (3)	−0.002 (4)
C8A	0.028 (3)	0.054 (4)	0.070 (5)	0.006 (3)	0.006 (3)	−0.001 (4)
C9A	0.041 (4)	0.041 (4)	0.077 (5)	0.016 (3)	0.010 (4)	0.005 (4)
C10A	0.038 (3)	0.053 (5)	0.036 (3)	0.000 (3)	−0.006 (2)	−0.008 (3)
C11A	0.050 (4)	0.061 (5)	0.031 (3)	−0.005 (3)	−0.002 (3)	0.008 (3)
C12A	0.051 (4)	0.034 (4)	0.050 (4)	0.006 (3)	−0.004 (3)	0.001 (3)
C13A	0.051 (4)	0.041 (4)	0.045 (4)	−0.017 (3)	0.004 (3)	0.009 (3)
C14A	0.032 (3)	0.034 (3)	0.038 (3)	−0.001 (2)	0.003 (2)	−0.009 (3)
C15A	0.042 (3)	0.041 (4)	0.052 (4)	−0.016 (3)	0.007 (3)	−0.008 (3)
C16A	0.040 (4)	0.061 (5)	0.051 (4)	−0.016 (3)	−0.006 (3)	−0.018 (4)
C17A	0.041 (4)	0.063 (5)	0.044 (4)	−0.004 (3)	−0.008 (3)	−0.005 (3)
C18A	0.040 (3)	0.045 (4)	0.039 (3)	−0.003 (3)	−0.008 (3)	0.001 (3)
Cl1B	0.039 (3)	0.043 (3)	0.044 (3)	−0.017 (3)	0.012 (3)	−0.007 (3)
O1B	0.069 (7)	0.078 (9)	0.099 (9)	0.024 (7)	0.016 (7)	0.012 (7)
O2B	0.099 (9)	0.086 (10)	0.061 (6)	0.018 (8)	0.015 (6)	0.016 (7)
O3B	0.053 (7)	0.059 (11)	0.082 (8)	−0.004 (7)	0.026 (6)	−0.011 (8)
O4B	0.098 (10)	0.097 (10)	0.099 (9)	−0.042 (8)	0.011 (8)	−0.043 (8)
Cl1C	0.047 (5)	0.053 (5)	0.049 (5)	0.010 (4)	0.006 (4)	0.014 (4)
O1C	0.059 (7)	0.121 (11)	0.087 (9)	−0.020 (8)	−0.029 (7)	0.031 (8)
O2C	0.088 (10)	0.081 (10)	0.069 (8)	0.004 (8)	0.031 (7)	−0.013 (8)
O3C	0.050 (8)	0.046 (10)	0.073 (8)	−0.021 (8)	0.005 (8)	−0.014 (8)
O4C	0.075 (9)	0.046 (7)	0.088 (9)	−0.005 (7)	0.021 (7)	−0.009 (7)

### {1,4-Bis[2-(pyridin-2-yl)ethyl]piperazine}chloridocobalt(II) perchlorate (ta-eab1701-c) Geometric parameters (Å, º)

**Table:**

Co1A—N1A	2.057 (5)	C8A—H8AB	0.9700
Co1A—N3A	2.099 (5)	C9A—H9AA	0.9700
Co1A—N4A	2.109 (5)	C9A—H9AB	0.9700
Co1A—N2A	2.236 (5)	C10A—C11A	1.526 (11)
Co1A—Cl1A	2.2780 (16)	C10A—H10A	0.9700
N1A—C1A	1.344 (8)	C10A—H10B	0.9700
N1A—C5A	1.366 (8)	C11A—H11A	0.9700
N2A—C7A	1.467 (10)	C11A—H11B	0.9700
N2A—C10A	1.468 (8)	C12A—C13A	1.538 (10)
N2A—C8A	1.476 (9)	C12A—H12A	0.9700
N3A—C12A	1.470 (9)	C12A—H12B	0.9700
N3A—C11A	1.480 (8)	C13A—C14A	1.504 (9)
N3A—C9A	1.500 (9)	C13A—H13A	0.9700
N4A—C14A	1.344 (9)	C13A—H13B	0.9700
N4A—C18A	1.352 (8)	C14A—C15A	1.401 (9)
C1A—C2A	1.372 (10)	C15A—C16A	1.368 (12)
C1A—H1A	0.9300	C15A—H15A	0.9300
C2A—C3A	1.363 (11)	C16A—C17A	1.348 (12)
C2A—H2A	0.9300	C16A—H16A	0.9300
C3A—C4A	1.387 (11)	C17A—C18A	1.386 (9)
C3A—H3A	0.9300	C17A—H17A	0.9300
C4A—C5A	1.376 (10)	C18A—H18A	0.9300
C4A—H4A	0.9300	Cl1B—O4B	1.400 (13)
C5A—C6A	1.506 (9)	Cl1B—O1B	1.401 (13)
C6A—C7A	1.537 (10)	Cl1B—O2B	1.409 (13)
C6A—H6AA	0.9700	Cl1B—O3B	1.442 (13)
C6A—H6AB	0.9700	Cl1C—O1C	1.395 (14)
C7A—H7AA	0.9700	Cl1C—O2C	1.400 (15)
C7A—H7AB	0.9700	Cl1C—O4C	1.417 (15)
C8A—C9A	1.535 (11)	Cl1C—O3C	1.425 (15)
C8A—H8AA	0.9700		
			
N1A—Co1A—N3A	123.7 (2)	C9A—C8A—H8AB	110.0
N1A—Co1A—N4A	100.7 (2)	H8AA—C8A—H8AB	108.3
N3A—Co1A—N4A	94.3 (2)	N3A—C9A—C8A	107.9 (6)
N1A—Co1A—N2A	84.2 (2)	N3A—C9A—H9AA	110.1
N3A—Co1A—N2A	69.5 (2)	C8A—C9A—H9AA	110.1
N4A—Co1A—N2A	162.6 (2)	N3A—C9A—H9AB	110.1
N1A—Co1A—Cl1A	115.11 (16)	C8A—C9A—H9AB	110.1
N3A—Co1A—Cl1A	115.81 (17)	H9AA—C9A—H9AB	108.4
N4A—Co1A—Cl1A	98.25 (16)	N2A—C10A—C11A	108.5 (5)
N2A—Co1A—Cl1A	94.62 (15)	N2A—C10A—H10A	110.0
C1A—N1A—C5A	117.7 (6)	C11A—C10A—H10A	110.0
C1A—N1A—Co1A	120.5 (4)	N2A—C10A—H10B	110.0
C5A—N1A—Co1A	121.4 (4)	C11A—C10A—H10B	110.0
C7A—N2A—C10A	112.4 (6)	H10A—C10A—H10B	108.4
C7A—N2A—C8A	113.8 (6)	N3A—C11A—C10A	108.8 (5)
C10A—N2A—C8A	107.3 (6)	N3A—C11A—H11A	109.9
C7A—N2A—Co1A	117.8 (4)	C10A—C11A—H11A	109.9
C10A—N2A—Co1A	101.5 (3)	N3A—C11A—H11B	109.9
C8A—N2A—Co1A	102.7 (4)	C10A—C11A—H11B	109.9
C12A—N3A—C11A	112.3 (6)	H11A—C11A—H11B	108.3
C12A—N3A—C9A	111.1 (6)	N3A—C12A—C13A	112.2 (6)
C11A—N3A—C9A	107.0 (6)	N3A—C12A—H12A	109.2
C12A—N3A—Co1A	116.8 (4)	C13A—C12A—H12A	109.2
C11A—N3A—Co1A	106.5 (4)	N3A—C12A—H12B	109.2
C9A—N3A—Co1A	102.2 (4)	C13A—C12A—H12B	109.2
C14A—N4A—C18A	118.3 (5)	H12A—C12A—H12B	107.9
C14A—N4A—Co1A	124.6 (4)	C14A—C13A—C12A	113.8 (6)
C18A—N4A—Co1A	116.6 (4)	C14A—C13A—H13A	108.8
N1A—C1A—C2A	123.2 (6)	C12A—C13A—H13A	108.8
N1A—C1A—H1A	118.4	C14A—C13A—H13B	108.8
C2A—C1A—H1A	118.4	C12A—C13A—H13B	108.8
C3A—C2A—C1A	119.1 (7)	H13A—C13A—H13B	107.7
C3A—C2A—H2A	120.5	N4A—C14A—C15A	120.5 (6)
C1A—C2A—H2A	120.5	N4A—C14A—C13A	118.6 (5)
C2A—C3A—C4A	119.0 (7)	C15A—C14A—C13A	120.8 (6)
C2A—C3A—H3A	120.5	C16A—C15A—C14A	119.6 (7)
C4A—C3A—H3A	120.5	C16A—C15A—H15A	120.2
C5A—C4A—C3A	119.9 (7)	C14A—C15A—H15A	120.2
C5A—C4A—H4A	120.0	C17A—C16A—C15A	120.4 (6)
C3A—C4A—H4A	120.0	C17A—C16A—H16A	119.8
N1A—C5A—C4A	121.0 (6)	C15A—C16A—H16A	119.8
N1A—C5A—C6A	117.4 (6)	C16A—C17A—C18A	118.1 (7)
C4A—C5A—C6A	121.6 (6)	C16A—C17A—H17A	120.9
C5A—C6A—C7A	113.7 (6)	C18A—C17A—H17A	120.9
C5A—C6A—H6AA	108.8	N4A—C18A—C17A	123.0 (7)
C7A—C6A—H6AA	108.8	N4A—C18A—H18A	118.5
C5A—C6A—H6AB	108.8	C17A—C18A—H18A	118.5
C7A—C6A—H6AB	108.8	O4B—Cl1B—O1B	106.3 (12)
H6AA—C6A—H6AB	107.7	O4B—Cl1B—O2B	114.9 (14)
N2A—C7A—C6A	112.4 (5)	O1B—Cl1B—O2B	108.5 (11)
N2A—C7A—H7AA	109.1	O4B—Cl1B—O3B	106.6 (13)
C6A—C7A—H7AA	109.1	O1B—Cl1B—O3B	112.3 (13)
N2A—C7A—H7AB	109.1	O2B—Cl1B—O3B	108.4 (14)
C6A—C7A—H7AB	109.1	O1C—Cl1C—O2C	107.2 (15)
H7AA—C7A—H7AB	107.9	O1C—Cl1C—O4C	104.1 (15)
N2A—C8A—C9A	108.6 (5)	O2C—Cl1C—O4C	110.1 (13)
N2A—C8A—H8AA	110.0	O1C—Cl1C—O3C	108.3 (15)
C9A—C8A—H8AA	110.0	O2C—Cl1C—O3C	111.6 (15)
N2A—C8A—H8AB	110.0	O4C—Cl1C—O3C	115.0 (15)

### {1,4-Bis[2-(pyridin-2-yl)ethyl]piperazine}chloridocobalt(II) perchlorate (ta-eab1701-c) Hydrogen-bond geometry (Å, º)

**Table:**

*D*—H···*A*	*D*—H	H···*A*	*D*···*A*	*D*—H···*A*
C2*A*—H2*A*···O4*B*^i^	0.93	2.76	3.454 (19)	133
C2*A*—H2*A*···O4*C*^i^	0.93	2.63	3.439 (18)	146
C7*A*—H7*AA*···O4*C*^ii^	0.97	2.49	3.34 (2)	146
C10*A*—H10*B*···O3*B*	0.97	2.60	3.30 (2)	129
C11*A*—H11*A*···O3*B*	0.97	2.54	3.28 (2)	133
C12*A*—H12*B*···O3*B*^iii^	0.97	2.67	3.53 (3)	147
C13*A*—H13*A*···O4*B*	0.97	2.54	3.461 (17)	159
C17*A*—H17*A*···O2*B*^iv^	0.93	2.68	3.271 (17)	122

Symmetry codes: (i) −*x*+2, *y*−1/2, −*z*+1; (ii) −*x*+1, *y*−1/2, −*z*+1; (iii) −*x*+1, *y*+1/2, −*z*+1; (iv) *x*, *y*, *z*+1.

### Dichlorido{4-methyl-1-[2-(pyridin-2-yl)ethyl]-1,4-diazacycloheptane}cobalt(II) (ta-eab1607) Crystal data

**Table:**

[CoCl_2_(C_13_H_21_N_3_)]	*F*(000) = 724
*M**_r_* = 349.16	*D*_x_ = 1.485 Mg m^−^^3^
Monoclinic, *P*2_1_/*n*	Cu *K*α radiation, λ = 1.54184 Å
*a* = 10.3626 (6) Å	Cell parameters from 1666 reflections
*b* = 11.5871 (7) Å	θ = 3.4–70.8°
*c* = 13.7035 (7) Å	µ = 11.67 mm^−^^1^
β = 108.308 (6)°	*T* = 273 K
*V* = 1562.12 (16) Å^3^	Needle, violet
*Z* = 4	0.42 × 0.08 × 0.06 mm

### Dichlorido{4-methyl-1-[2-(pyridin-2-yl)ethyl]-1,4-diazacycloheptane}cobalt(II) (ta-eab1607) Data collection

**Table:**

Rigaku-OxfordDiffracti0on diffractometer	2957 independent reflections
Radiation source: fine-focus sealed X-ray tube, Enhance (Cu) X-ray Source	1805 reflections with *I* > 2σ(*I*)
Graphite monochromator	*R*_int_ = 0.054
Detector resolution: 16.0416 pixels mm^-1^	θ_max_ = 71.4°, θ_min_ = 5.1°
ω scans	*h* = −11→12
Absorption correction: multi-scan (CrysAlisPro; Rigaku OD, 2015)	*k* = −9→14
*T*_min_ = 0.202, *T*_max_ = 1.000	*l* = −16→15
5711 measured reflections	

### Dichlorido{4-methyl-1-[2-(pyridin-2-yl)ethyl]-1,4-diazacycloheptane}cobalt(II) (ta-eab1607) Refinement

**Table:**

Refinement on *F*^2^	Primary atom site location: dual
Least-squares matrix: full	Hydrogen site location: inferred from neighbouring sites
*R*[*F*^2^ > 2σ(*F*^2^)] = 0.056	H-atom parameters constrained
*wR*(*F*^2^) = 0.139	*w* = 1/[σ^2^(*F*_o_^2^) + (0.0523*P*)^2^] where *P* = (*F*_o_^2^ + 2*F*_c_^2^)/3
*S* = 1.03	(Δ/σ)_max_ < 0.001
2957 reflections	Δρ_max_ = 0.54 e Å^−^^3^
173 parameters	Δρ_min_ = −0.33 e Å^−^^3^
0 restraints	

### Dichlorido{4-methyl-1-[2-(pyridin-2-yl)ethyl]-1,4-diazacycloheptane}cobalt(II) (ta-eab1607) Special details

**Table:**

Geometry. All esds (except the esd in the dihedral angle between two l.s. planes) are estimated using the full covariance matrix. The cell esds are taken into account individually in the estimation of esds in distances, angles and torsion angles; correlations between esds in cell parameters are only used when they are defined by crystal symmetry. An approximate (isotropic) treatment of cell esds is used for estimating esds involving l.s. planes.

### Dichlorido{4-methyl-1-[2-(pyridin-2-yl)ethyl]-1,4-diazacycloheptane}cobalt(II) (ta-eab1607) Fractional atomic coordinates and isotropic or equivalent isotropic displacement parameters (Å^2^)

**Table:**

	*x*	*y*	*z*	*U*_iso_*/*U*_eq_	
Co1	0.47418 (8)	0.55685 (8)	0.71591 (6)	0.0360 (2)	
Cl1	0.42833 (17)	0.63048 (14)	0.85722 (11)	0.0587 (4)	
Cl2	0.69468 (13)	0.55827 (16)	0.71540 (11)	0.0580 (4)	
N1	0.4898 (6)	0.3748 (4)	0.7718 (4)	0.0565 (13)	
N2	0.2983 (5)	0.4751 (4)	0.6221 (4)	0.0488 (12)	
N3	0.4278 (4)	0.7156 (4)	0.6315 (3)	0.0416 (10)	
C1	0.5068 (6)	0.8045 (5)	0.6743 (4)	0.0507 (15)	
H1	0.5807	0.7900	0.7324	0.061*	
C2	0.4869 (7)	0.9154 (5)	0.6387 (6)	0.0643 (18)	
H2	0.5442	0.9746	0.6726	0.077*	
C3	0.3805 (7)	0.9368 (6)	0.5522 (6)	0.0688 (18)	
H3	0.3634	1.0112	0.5259	0.083*	
C4	0.2996 (7)	0.8472 (5)	0.5049 (4)	0.0566 (16)	
H4	0.2275	0.8601	0.4453	0.068*	
C5	0.3249 (5)	0.7366 (5)	0.5455 (4)	0.0421 (12)	
C6	0.2368 (6)	0.6382 (6)	0.4937 (4)	0.0622 (18)	
H6A	0.1561	0.6695	0.4435	0.075*	
H6B	0.2854	0.5938	0.4564	0.075*	
C7	0.1931 (6)	0.5584 (6)	0.5622 (5)	0.0633 (17)	
H7A	0.1154	0.5148	0.5206	0.076*	
H7B	0.1631	0.6042	0.6102	0.076*	
C8	0.3381 (7)	0.3961 (6)	0.5516 (5)	0.0665 (19)	
H8A	0.4005	0.4366	0.5237	0.080*	
H8B	0.2576	0.3778	0.4947	0.080*	
C9	0.4033 (8)	0.2856 (6)	0.5980 (6)	0.075 (2)	
H9A	0.4372	0.2469	0.5482	0.090*	
H9B	0.3339	0.2366	0.6099	0.090*	
C10	0.5177 (7)	0.2958 (6)	0.6966 (5)	0.0635 (18)	
H10A	0.5376	0.2198	0.7274	0.076*	
H10B	0.5980	0.3225	0.6815	0.076*	
C11	0.2414 (7)	0.4125 (6)	0.6920 (6)	0.071 (2)	
H11A	0.1779	0.3548	0.6535	0.085*	
H11B	0.1917	0.4660	0.7213	0.085*	
C12	0.3524 (8)	0.3533 (6)	0.7791 (6)	0.078 (2)	
H12A	0.3482	0.3815	0.8447	0.094*	
H12B	0.3356	0.2708	0.7764	0.094*	
C13	0.5948 (8)	0.3600 (6)	0.8732 (5)	0.086 (3)	
H13A	0.6829	0.3747	0.8669	0.130*	
H13B	0.5915	0.2825	0.8970	0.130*	
H13C	0.5779	0.4132	0.9216	0.130*	

### Dichlorido{4-methyl-1-[2-(pyridin-2-yl)ethyl]-1,4-diazacycloheptane}cobalt(II) (ta-eab1607) Atomic displacement parameters (Å^2^)

**Table:**

	*U*^11^	*U*^22^	*U*^33^	*U*^12^	*U*^13^	*U*^23^
Co1	0.0361 (4)	0.0358 (4)	0.0344 (4)	0.0004 (4)	0.0086 (3)	0.0003 (4)
Cl1	0.0794 (10)	0.0529 (9)	0.0478 (8)	0.0211 (8)	0.0258 (7)	−0.0003 (7)
Cl2	0.0374 (6)	0.0691 (10)	0.0646 (9)	0.0021 (7)	0.0119 (6)	−0.0030 (8)
N1	0.083 (4)	0.040 (3)	0.052 (3)	0.011 (3)	0.029 (3)	0.010 (2)
N2	0.045 (2)	0.034 (2)	0.064 (3)	−0.006 (2)	0.013 (2)	−0.007 (2)
N3	0.040 (2)	0.040 (2)	0.039 (2)	−0.003 (2)	0.0042 (18)	0.008 (2)
C1	0.047 (3)	0.047 (3)	0.050 (3)	−0.012 (3)	0.004 (3)	0.005 (3)
C2	0.065 (4)	0.041 (4)	0.090 (5)	−0.011 (3)	0.029 (4)	0.000 (3)
C3	0.072 (4)	0.044 (4)	0.093 (5)	0.005 (4)	0.030 (4)	0.022 (4)
C4	0.062 (4)	0.052 (4)	0.051 (3)	0.013 (3)	0.009 (3)	0.015 (3)
C5	0.042 (3)	0.044 (3)	0.037 (3)	0.005 (3)	0.007 (2)	0.001 (2)
C6	0.059 (4)	0.058 (4)	0.050 (3)	0.010 (3)	−0.010 (3)	−0.004 (3)
C7	0.040 (3)	0.055 (4)	0.085 (5)	−0.007 (3)	0.005 (3)	−0.009 (4)
C8	0.079 (5)	0.055 (4)	0.060 (4)	−0.015 (4)	0.013 (3)	−0.015 (3)
C9	0.090 (5)	0.055 (4)	0.089 (5)	−0.001 (4)	0.041 (4)	−0.022 (4)
C10	0.079 (5)	0.043 (4)	0.072 (4)	0.020 (3)	0.030 (4)	0.006 (3)
C11	0.064 (4)	0.043 (4)	0.123 (6)	−0.013 (3)	0.055 (4)	−0.001 (4)
C12	0.124 (6)	0.042 (4)	0.094 (5)	−0.010 (4)	0.071 (5)	0.019 (4)
C13	0.125 (7)	0.068 (5)	0.063 (4)	0.032 (5)	0.024 (4)	0.033 (4)

### Dichlorido{4-methyl-1-[2-(pyridin-2-yl)ethyl]-1,4-diazacycloheptane}cobalt(II) (ta-eab1607) Geometric parameters (Å, º)

**Table:**

Co1—Cl1	2.2981 (16)	C6—H6A	0.9700
Co1—Cl2	2.2872 (15)	C6—H6B	0.9700
Co1—N1	2.232 (5)	C6—C7	1.486 (9)
Co1—N2	2.097 (4)	C7—H7A	0.9700
Co1—N3	2.146 (4)	C7—H7B	0.9700
N1—C10	1.473 (8)	C8—H8A	0.9700
N1—C12	1.479 (9)	C8—H8B	0.9700
N1—C13	1.482 (8)	C8—C9	1.493 (9)
N2—C7	1.494 (7)	C9—H9A	0.9700
N2—C8	1.480 (8)	C9—H9B	0.9700
N2—C11	1.465 (8)	C9—C10	1.496 (9)
N3—C1	1.331 (7)	C10—H10A	0.9700
N3—C5	1.340 (6)	C10—H10B	0.9700
C1—H1	0.9300	C11—H11A	0.9700
C1—C2	1.367 (8)	C11—H11B	0.9700
C2—H2	0.9300	C11—C12	1.535 (10)
C2—C3	1.365 (9)	C12—H12A	0.9700
C3—H3	0.9300	C12—H12B	0.9700
C3—C4	1.363 (9)	C13—H13A	0.9600
C4—H4	0.9300	C13—H13B	0.9600
C4—C5	1.389 (8)	C13—H13C	0.9600
C5—C6	1.493 (8)		
			
Cl2—Co1—Cl1	118.10 (7)	C7—C6—H6A	108.3
N1—Co1—Cl1	94.21 (14)	C7—C6—H6B	108.3
N1—Co1—Cl2	92.47 (15)	N2—C7—H7A	108.3
N2—Co1—Cl1	108.33 (15)	N2—C7—H7B	108.3
N2—Co1—Cl2	132.67 (15)	C6—C7—N2	115.8 (5)
N2—Co1—N1	74.86 (19)	C6—C7—H7A	108.3
N2—Co1—N3	93.00 (17)	C6—C7—H7B	108.3
N3—Co1—Cl1	93.75 (13)	H7A—C7—H7B	107.4
N3—Co1—Cl2	92.70 (13)	N2—C8—H8A	108.4
N3—Co1—N1	167.11 (18)	N2—C8—H8B	108.4
C10—N1—Co1	110.9 (4)	N2—C8—C9	115.7 (6)
C10—N1—C12	110.3 (5)	H8A—C8—H8B	107.4
C10—N1—C13	109.6 (5)	C9—C8—H8A	108.4
C12—N1—Co1	102.4 (4)	C9—C8—H8B	108.4
C12—N1—C13	110.8 (6)	C8—C9—H9A	108.2
C13—N1—Co1	112.6 (4)	C8—C9—H9B	108.2
C7—N2—Co1	112.9 (3)	C8—C9—C10	116.2 (6)
C8—N2—Co1	108.2 (4)	H9A—C9—H9B	107.4
C8—N2—C7	110.2 (5)	C10—C9—H9A	108.2
C11—N2—Co1	105.9 (4)	C10—C9—H9B	108.2
C11—N2—C7	107.8 (5)	N1—C10—C9	114.0 (5)
C11—N2—C8	111.8 (5)	N1—C10—H10A	108.8
C1—N3—Co1	115.0 (3)	N1—C10—H10B	108.8
C1—N3—C5	117.2 (5)	C9—C10—H10A	108.8
C5—N3—Co1	127.7 (4)	C9—C10—H10B	108.8
N3—C1—H1	117.7	H10A—C10—H10B	107.6
N3—C1—C2	124.5 (5)	N2—C11—H11A	109.2
C2—C1—H1	117.7	N2—C11—H11B	109.2
C1—C2—H2	121.0	N2—C11—C12	111.9 (5)
C3—C2—C1	118.1 (6)	H11A—C11—H11B	107.9
C3—C2—H2	121.0	C12—C11—H11A	109.2
C2—C3—H3	120.6	C12—C11—H11B	109.2
C4—C3—C2	118.9 (6)	N1—C12—C11	111.9 (5)
C4—C3—H3	120.6	N1—C12—H12A	109.2
C3—C4—H4	119.9	N1—C12—H12B	109.2
C3—C4—C5	120.1 (5)	C11—C12—H12A	109.2
C5—C4—H4	119.9	C11—C12—H12B	109.2
N3—C5—C4	121.2 (5)	H12A—C12—H12B	107.9
N3—C5—C6	118.6 (5)	N1—C13—H13A	109.5
C4—C5—C6	120.2 (5)	N1—C13—H13B	109.5
C5—C6—H6A	108.3	N1—C13—H13C	109.5
C5—C6—H6B	108.3	H13A—C13—H13B	109.5
H6A—C6—H6B	107.4	H13A—C13—H13C	109.5
C7—C6—C5	115.9 (5)	H13B—C13—H13C	109.5
